# Spectral Composition of Light Affects Sensitivity to UV-B and Photoinhibition in Cucumber

**DOI:** 10.3389/fpls.2020.610011

**Published:** 2021-01-05

**Authors:** Carolina Falcato Fialho Palma, Victor Castro-Alves, Luis Orlando Morales, Eva Rosenqvist, Carl-Otto Ottosen, Åke Strid

**Affiliations:** ^1^Department of Food Science, Plant, Food & Climate, Aarhus University, Aarhus, Denmark; ^2^School of Science and Technology, Örebro Life Science Centre, Örebro University, Örebro, Sweden; ^3^Department of Plant and Environmental Sciences, Crop Sciences, University of Copenhagen, Taastrup, Denmark

**Keywords:** UV-B, LEDs, light quality, chlorophyll fluorescence, gas exchange, cucumber, morphology

## Abstract

Ultraviolet B (UV-B) (280–315 nm) and ultraviolet A (UV-A) (315–400 nm) radiation comprise small portions of the solar radiation but regulate many aspects of plant development, physiology and metabolism. Until now, how plants respond to UV-B in the presence of different light qualities is poorly understood. This study aimed to assess the effects of a low UV-B dose (0.912 ± 0.074 kJ m^–2^ day^–1^, at a 6 h daily UV exposure) in combination with four light treatments (blue, green, red and broadband white at 210 μmol m^–2^ s^–1^ Photosynthetically active radiation [PAR]) on morphological and physiological responses of cucumber (*Cucumis sativus* cv. “Lausanna RZ F1”). We explored the effects of light quality backgrounds on plant morphology, leaf gas exchange, chlorophyll fluorescence, epidermal pigment accumulation, and on acclimation ability to saturating light intensity. Our results showed that supplementary UV-B significantly decreased biomass accumulation in the presence of broad band white, blue and green light, but not under red light. UV-B also reduced the photosynthetic efficiency of CO_2_ fixation (α) when combined with blue light. These plants, despite showing high accumulation of anthocyanins, were unable to cope with saturating light conditions. No significant effects of UV-B in combination with green light were observed for gas exchange and chlorophyll fluorescence parameters, but supplementary UV-B significantly increased chlorophyll and flavonol contents in the leaf epidermis. Plants grown under red light and UV-B significantly increased maximum photosynthetic rate and dark respiration compared to pure red light. Additionally, red and UV-B treated plants exposed to saturating light intensity showed higher quantum yield of photosystem II (PSII), fraction of open PSII centres and electron transport rate and showed no effect on the apparent maximum quantum efficiency of PSII photochemistry (F_v_/F_m_) or non-photochemical quenching, in contrast to solely red-light conditions. These findings provide new insights into how plants respond to UV-B radiation in the presence of different light spectra.

## Introduction

Plants perceive signals from their surrounding environment and regulate their growth and development accordingly (Smith, 1982; Huché-Thélier et al., 2016). Plants are highly sensitive to the spectral distribution of light and perceive changes in the light spectra and intensity through several protein photoreceptors (Fankhauser and Chory, 1997). These photoreceptors are sensitive to specific regions of the spectrum and overlap of action spectra of different plant photoreceptors occur, allowing the plant to detect a wider and more complex range of changes in their light environment (Heijde and Ulm, 2012). Cryptochromes and phototropins are sensitive to blue light (400–500 nm) and ultraviolet (UV) radiation A (UV-A) (315–400 nm), whereas phytochromes perceive red (600–700 nm) and far-red (700–800 nm) light. Moreover, phytochromes and cryptochromes also absorb light in the green wavelength region (500–600 nm) (Folta and Maruhnich, 2007), although the response of these photoreceptors to green light is extremely weak compared to red and blue radiation, respectively.

Ultraviolet B (UV-B) radiation (290–315 nm) comprises a small but energetic portion of the solar radiation that also reaches the surface of the Earth. UV-B perceived through the photoreceptor UV RESISTANCE LOCUS 8 (UVR8) (Rizzini et al., 2011) largely affects plant morphology and metabolism (Jenkins, 2017). Plant responses to UV-B radiation are highly dependent on UV-B dosage and are also affected by whether or not the plants have previously been UV-B acclimated (Huché-Thélier et al., 2016; Jenkins, 2017), as well as by the accumulation of photosynthetic pigments and phenolic compounds in the leaf epidermis. In addition, the levels of the photosynthetically active radiation (PAR) (400–700 nm) and the PAR/UV-B ratio are factors that strongly influence plant UV-B responses (Krizek, 2004; Lidon et al., 2012; Jenkins, 2017).

Exposure to high doses of UV-B may also induce (di)stress responses in plants, triggering the formation of free radicals [reactive oxygen species (ROS)] that cause oxidative damage (Day and Vogelmann, 1995; Jansen et al., 1998; Hideg et al., 2013). Plant responses to UV-B are highly species specific and morphological responses can be either positive (increase in plant growth) or negative (decrease in plant growth) (Huché-Thélier et al., 2016). UV-B can also reduce stem extension and leaf expansion and affect leaf thickness, leaf curling and auxiliary branching (Jansen, 2002; Wargent et al., 2009; Klem et al., 2012; Jenkins, 2017; Qian et al., 2020). Moreover, under low doses, UV-B radiation can promote the accumulation of photoprotective compounds in the leaf tissue (Day et al., 1993). For instance, an increase in the accumulation of flavonoid glycosides in response to UV-B has been described both under artificial and natural conditions (Krizek et al., 1997; Demkura and Ballaré, 2012; Zhao et al., 2020), although in some instances UV-B had no effect or even led to decreased flavonoid accumulation (Huché-Thélier et al., 2016). Flavonoids, particularly anthocyanins, are mainly accumulated in vacuoles in the upper layer of the leaf epidermis although they can also be found in the cell wall, chloroplast envelope and cell nucleus (Hideg and Strid, 2017). Apart from having a strong free-radical scavenging activity (Lattanzio et al., 2006; Agati and Tattini, 2010), flavonoids absorb radiation in the UV range of the spectrum (280–340 nm), functioning as sunscreen compounds to protect plants from further UV induced damage (Day et al., 1993).

Contradicting results in UV-B research often derive from methodological differences among studies or from species or ecotype differences (Kalbina and Strid, 2006). Different UV-B doses, light environments (natural sunlight vs. artificial lighting), other abiotic factors and species-specific responses cause variation between studies. Additionally, most UV-B research has been performed using broadband white light background under controlled conditions. Hence, there is a lack of studies depicting the effects of UV-B radiation on whole plant responses under monochromatic light backgrounds. This type of research is important to assess plant responses triggered by crosstalk between different light qualities and their impact on plant growth and development. With the development of light emitting diode (LED) technology, the use of LED lighting for horticultural production is increasing. Because of the high energy efficiency, customizable light environment and low radiant heat that allows for the placement of the lamps close to the canopy (Bourget, 2008; Darko et al., 2014; Singh et al., 2015), an interest in the use of LED lights in multilayer production has emerged. Multilayer systems allow the production of the same number of plants in a smaller area and could be relevant to use for intensive production systems such as germination of seedlings or rooting of cuttings. These production systems, although not yet economically feasible in all geographical regions when compared to normal greenhouse production (Graamans et al., 2018), rely on the sole use of LEDs and provide a unique environment for investigating new opportunities of LED lighting use, such as in monochromatic illumination and the use of UV to manipulate plant growth and development.

Blue light perception is often involved in physiological processes such as photomorphogenesis, phototropism (de Carbonnel et al., 2010), stomatal opening (Briggs and Huala, 1999; Boccalandro et al., 2012) and chlorophyll formation. Moreover, blue light induces an accumulation of several phytonutrients in the leaves, such as phenolic acids and flavonoids (Ohashi-Kaneko et al., 2007; Li and Kubota, 2009; Nascimento et al., 2013; Ouzounis et al., 2014, 2015). Red light can regulate vegetative development and plant architecture by influencing phototropism and shade-avoidance syndrome (SAS) (Fankhauser and Chory, 1997; Demotes-Mainard et al., 2016) and promote the accumulation of anthocyanins in the leaf epidermis (Mizuno et al., 2011; Zoratti et al., 2014; Garrett Owen and Lopez, 2015). Green light can inhibit stomatal opening stimulated by blue light (Frechilla et al., 2000; Smith et al., 2017) and promote early stem elongation (Folta, 2004). These observations suggest that different monochromatic light spectra not only have a different impact on plant growth, but could also influence plants ability to cope with abiotic stress (e.g., high light) due to a wavelength-driven accumulation of photoprotective compounds (Bayat et al., 2018).

Cucumber (*Cucumis sativus* L.), is an important food crop with fast growth and high sensitivity toward the spectral composition of the light environment. These aspects make cucumber an interesting crop for studying light-driven responses in plants, such as responses to UV radiation (Qian et al., 2019, 2020). The aim of this study was to investigate the effects of supplementary UV-B on growth, morphology and physiology of cucumber plants grown under different monochromatic light backgrounds. We hypothesized that: (I) different monochromatic lights have different impacts on plant morphology and (II) the response of cucumber to UV-B radiation is highly dependent on individual monochromatic light backgrounds.

## Materials and Methods

### Plant Material and Growing Conditions

Cucumber seeds (*cv*. “Lausanna RZ F1,” Semenco, Asmundtorp, Sweden) were individually sown in 8 × 8 cm pots filled with peat substrate (Grön Torvmull 50-liter, SW Horto, Hasselfors Garden, Örebro, Sweden). The seeds were germinated under artificial light at 200 μmol m^–2^ s^–1^ PAR provided by metal halide lamps (MASTER HPI-T Plus 400 W/645, Phillips) during a 16 h photoperiod (6:00 to 22:00). The germination took place at room temperature of 22 ± 1/18 ± 1°C day/night and 60 ± 5% relative humidity. Immediately after germination and opening of the cotyledons, the seedlings were randomly transferred to a room without natural light and placed in four 2 m high custom-made trolleys with an 80 × 170 cm ebb/flow watering table. Each trolley had a unique light spectral treatment (four trolleys in total) and contained 72 treatment plants. These plants were later randomly divided into 36 control plants per treatment that were only exposed to one of four different light spectra in the PAR region and 36 plants that in addition to the different light spectra were exposed to low levels of UV-B (for both treatment types, see description below). Plants remained under four different LED light treatments for 23 days at a constant light intensity of 200–215 μmol m^–2^ s^–1^ PAR and 16 h photoperiod (6:00 to 22:00). The climate conditions in the room were maintained at 22 ± 1/18 ± 1°C day/night and RH of 60 ± 5%. No external supply of carbon dioxide (CO_2_) was used. The cucumber plants were watered daily by flood irrigation containing commercial mineral nutrient solution (composition: 3.1 g NO_3_^–^, 2.0 g NH_4_^+^, 1.0 g P, 4.3 g K, 0.4 g S, 0.3 g Ca, 0.4 g Mg, 35 mg Fe, 20 mg Mn, 10 mg B, 3.0 mg Zn, 1.5 mg Cu, 0.4 mg Mo; pH 6.5 and EC 1.4 mS cm^–1^; Blomstra växtnäring, Orkla, Solna, Sweden). A portable data logger (Tinytag, Gemini Data Loggers Ltd., Chichester, United Kingdom) placed within the canopy recorded the temperature and relative humidity of each light treatment (Supplementary Figure S2).

### Light Treatments

From cotyledon stage until the final harvest, plants were grown under four light treatments created in the trolleys by using FL300 LED luminaires (Senmatic, Søndersø, Denmark). The white light was created by the commercially available white broadband FL300 Sunlight (33% blue [400–500 nm], 40% green [500–600 nm] and 27% red [600–700 nm]), while the monochromatic FL300 were custom made: blue (wavelength peak at 448 nm), green (528 nm), and red (660 nm). The lamps were adjusted to give 200–215 μmol m^–2^ s^–1^ PAR at plant height (Table 1), creating a daily light integral (DLI) of approx. 10.5 mol m^–2^ d^–1^. Because of the comparatively low photosynthetic photon flux efficacy from green LEDs the green FL300 lamp was complemented by two custom-made narrow, green luminaires (Fluence Bioengineering, Austin, TX, United States). To eliminate stray light the sides of the trolleys were covered with non-transparent black/white plastic with the white side facing inward. Additionally, the position effect within each treatment was minimized by randomizing the treatment pots daily.

**TABLE 1:** Photosynthetically active radiation of four different light backgrounds (broadband White, Blue, Green, and Red).

Light treatment	White	Blue	Green	Red
PAR [400–800 nm]	214	212	201/180^#^	211
(μmol m^–2^ s^–1^)				

*The values represent averages. ^#^Due to the extra green LEDs and the resulting architecture of the light equipment, the UV tube was hanging lower and partly shaded the green lights, thus giving lower PAR in the CA box than in the “Perspex only” box. This shading effect was not happening in the three other light qualities.*

**FIGURE 1:**
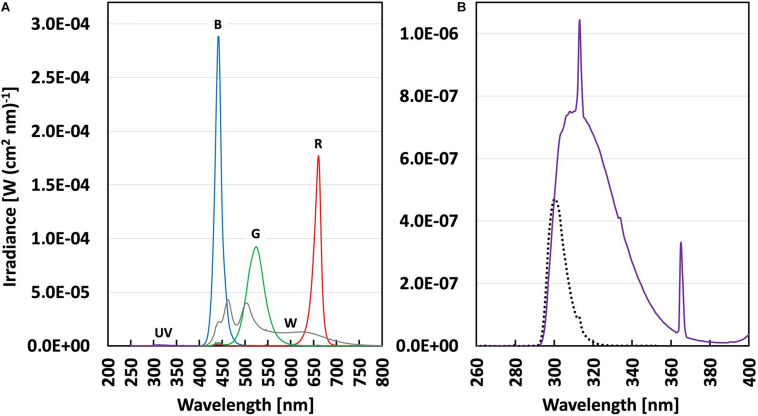
**(A)** Spectral irradiance (in W(cm^2^ nm)^–1^) in Perspex covered (control) and cellulose acetate covered (UV-B) boxes under four different PAR backgrounds (Table 1); broadband White (gray line), Blue, Green, Red (with lines of respective color) and UV-B (violet line). **(B)** Spectral irradiance in the UV range (violet line) with enlarged scale and the plant-weighted UV dose (black dotted line) in the UV-B treatments.

The cucumber seedlings were exposed to UV-B radiation 9 days after the start of the light treatments, when the first true leaf was fully expanded. Two open top, front and backside Perspex boxes (OTFB boxes; *c.f.* Qian et al., 2019) were used in each trolley to filter the UV radiation. The open top, front and backsides of the OTFB boxes were covered with sheets of Perspex to block all UV radiation for the exposure of control plants, while 0.13 mm cellulose diacetate (CA) sheets (Nordbergs Tekniska AB, Vallentuna, Sweden) were used for the UV-treated plants to block mainly UV-C radiation (<292 nm). The UV was provided by fluorescent tubes (Philips TL20/12 UV, Eindhoven, Netherlands). The spectra of both UV and the visible light were measured inside the OTFB boxes, with an OL756 double monochromator spectroradiometer (Optronic Laboratories, Orlando, FL, United States) with the orifice of the upward-directed integrating sphere placed approximately 20 cm above the table, at plant height (Figures 1A,B). The plant-weighted UV normalized to 300 nm (Thimijan et al., 1978; Yu and Björn, 1997; Kalbina et al., 2008) shows that the UV provided is biologically active in plants almost exclusively in the UV-B range (280–315 nm) (Figure 1B). The plant-weighted UV normalized to 300 nm was quantified to 42.4 ± 3.4 mW m^–2^, corresponding to 0.912 ± 0.074 kJ m^–2^ day^–1^ (at a 6 h daily UV exposure). Plants were exposed to UV-B radiation for 14 days. Thereafter 22 plants per treatment were measured and harvested (see below). To investigate whether the different light acclimation regimens induced a difference in the ability to cope with photoinhibition, the remaining treatment plants were subjected to a saturating light treatment for 5 h at 1600 μmol m^–2^ s^–1^ PAR provided by two FL300 Sunlight luminaires, delivering an additional light integral of 29 mol m^–2^ 5 h^–1^.

### Plant Growth and Development

Plant growth was assessed for 5–7 plants per treatment through destructive harvest at the end of the UV-B radiation treatment. The plant height was measured from the stem base to the apical meristem and the stem diameter (Ø) 1 cm above the soil using a digital slide caliper (Biltema, Linköping, Sweden). The number of true leaves and leaf area (LA) was measured on scanned leaves using the Image J software (version 1.52a) (Wayne Rasband, National Institute of Health, United States). After each harvest, leaf and stem fresh mass (LFM and SFM) and dry mass (LDM and SDM) were determined after drying for 3 days at 80°C. Specific leaf mass (SLM = leaf DM/LA), leaf mass ratio (LMR = leaf DM/total DM), individual leaf area (ILA = LA/leaf number), internode length (INL = height/leaf number) and dry mass per cent (DM% = total DM/total FM) were calculated.

### Gas Exchange Measurements

The photosynthetic CO_2_ assimilation rate (A_n_), transpiration rate (E), stomatal conductance (g_s_) and intracellular CO_2_ concentration (C_i_) were estimated in seven plants per treatment by gas exchange (CIRAS-2 with PLC6(U) with a LED light source, PP Systems, Amesbury, United States) on the last fully expanded leaf on day 9 to 12 of the UV-B treatment. The conditions during measurement were 22°C leaf temperature, 400 ppm CO_2_ and 0.9 ± 1.0 Pa kPa^–1^ vapor pressure deficit (VPD). The light response curve covered 12 light levels starting at a PAR of 250 μmol m^–2^ s^–1^, decreasing in steps to 20 μmol m^–2^ s^–1^ and again from 250 increasing in steps to 1800 μmol m^–2^ s^–1^. Data were logged every 5 s and the mean value of 1 min of steady-state was averaged for each light level. The light response curves were fitted by Solver in Excel to a non-rectangular hyperbola (Ögren, 1993) to determine plant dark respiration rate (R_dark_), the apparent quantum yield of CO_2_ assimilation based on incident light (α), the light compensation point (LCP), maximum net assimilation rate at light saturation (A_max_), and the convexity (θ) of the light response curve.

### Chlorophyll Fluorescence Measurements

Chlorophyll fluorescence was measured using a Mini-PAM with leaf clips (Walz, Effeltrich, Germany) on day 13 to 14 of UV-B radiation and after the subsequent saturating light treatment (1600 m^–2^ s ^–1^) that was used to induce photoinhibition. Randomized measurements were performed in the afternoon (13:00–16:00) in 4–5 biological replicates per treatment. The F_o_ and F_m_ were measured for maximum photochemical efficiency of PSII (F_v_/F_m_ = (F_m_–F_o_)/F_m_) after 30 min dark adaptation with aluminum foil on the last fully expanded leaf. The site of measurement was marked on the leaf and the plant was placed under saturating PAR (1600 ± 100 μmol m^–2^ s^–1^) from a KL 1500 electronic halogen lamp (SCHOTT AG Lighting and Imaging, Mainz, Germany) for 30 min to reach steady-state photosynthesis. Thereafter, the leaf clip was placed on the marked spot, F′ and Fm′ were measured and the operation efficiency of PSII (Fq′/Fm′), the electron transport rate (ETR), the fraction of open PSII centres (q_L_) and the non-photochemical quenching (NPQ) were calculated (Murchie and Lawson, 2013).

### Non-destructive Optical Absorbance Measurements

Non-destructive measurements of chlorophyll, flavonol and anthocyanin contents were assessed on the adaxial side of the last fully developed leaf with a Dualex+ (FORCE-A, Centre Universitaire Paris Sud, Cedex, France). The four replicates per treatment were measured immediately after the daily UV-B exposure.

### Statistical Analysis

All data analyzed was collected from three independent experiments. Statistical analyses were performed in R (version 3.3.1., R Core Development Team, 2017). Linear mixed effects models were fitted using the *lme* function in the *nlme* package (Pinheiro and Bates, 2000) and with experimental replicate as a random component. The effect of the different PAR backgrounds (White, Blue, Green, Red) on plant growth, morphology and physiology was assessed using ANOVA. In case significant differences were identified among treatments, contrasts between the four PAR backgrounds were fitted using the function *fit.contrasts* from the *gmodels* package (Warnes et al., 2018). The resulting *p*-values were adjusted using the function *p.adjust* (Holm, 1979) (Supplementary Table S3). Additionally, the effect of supplementary UV-B irradiation was tested solely within the same PAR background and no comparison between light backgrounds was made. Differences between control and UV-B plants within the same light environment were tested using linear mixed effects models with experimental replicate as a random factor. Differences between control and UV-B-treated plants were assessed using ANOVA (Supplementary Table S4).

## Results

### Monochromatic Light Qualities Within the PAR Spectrum Differentially Regulate Growth and Development

**FIGURE 2:**
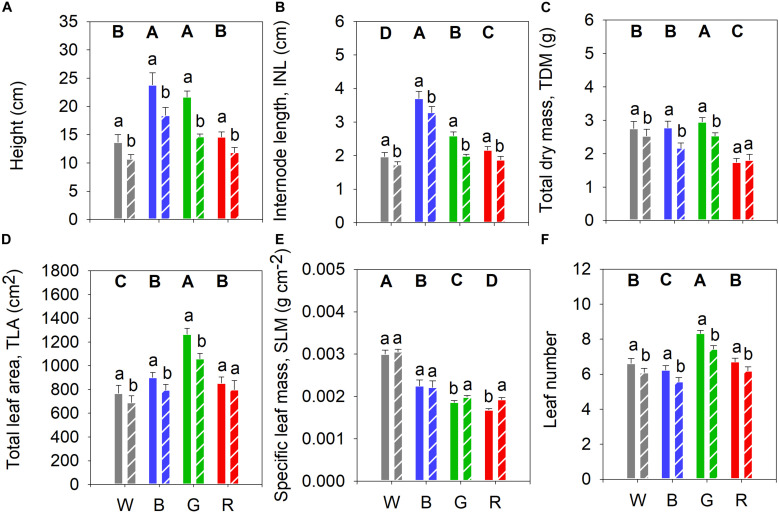
Biomass accumulation of cucumber plants grown under four PAR backgrounds after 14 days without (solid bars) or with (dashed bars) exposure to supplementary UV-B irradiation. **(A)** Height (cm); **(B)** Internode length (INL, cm); **(C)** Total dry mass (TDM, g); **(D)** Total leaf area (TLA, cm^2^); **(E)** Specific leaf mass (SLM, g cm^–2^); **(F)** Leaf number. Data are mean values (*n* = 21 ± SE). Capital letters indicate significant differences between growth light qualities without UV-B and lower-case letters between non-UV-B-exposed plants and UV-B-exposed plants within the same light backgrounds, both at *P* < 0.05.

To investigate the spectral effect on growth and development of cucumber plants, we analyzed non-UV-B (control plants) grown under different PAR spectra 24 days after germination (Figure 2). Plants grown under blue and green light were tallest compared to plants grown under the other light spectra (Figure 2A). INL followed the same pattern with decreasing length of internodes from blue to white growth light (Figure 2B). Plants grown under red light showed lower total dry mass (TDM) compared to plants grown under the other light spectra, whilst green light plants had the highest TDM (Figure 2C). Plants grown under green light had the largest total leaf area (TLA) whilst the plants under broadband white light had the smallest (Figure 2D). The white-light-grown plants had a higher SLM compared to plants grown under the other light spectra (Figure 2E). Plants grown under green light had the largest leaf number while blue-light plants had the smallest leaf number, compared with the other light treatments (Figure 2F).

### Green and Red Light Reduce Photosynthesis in Cucumber Leaves

**FIGURE 3:**
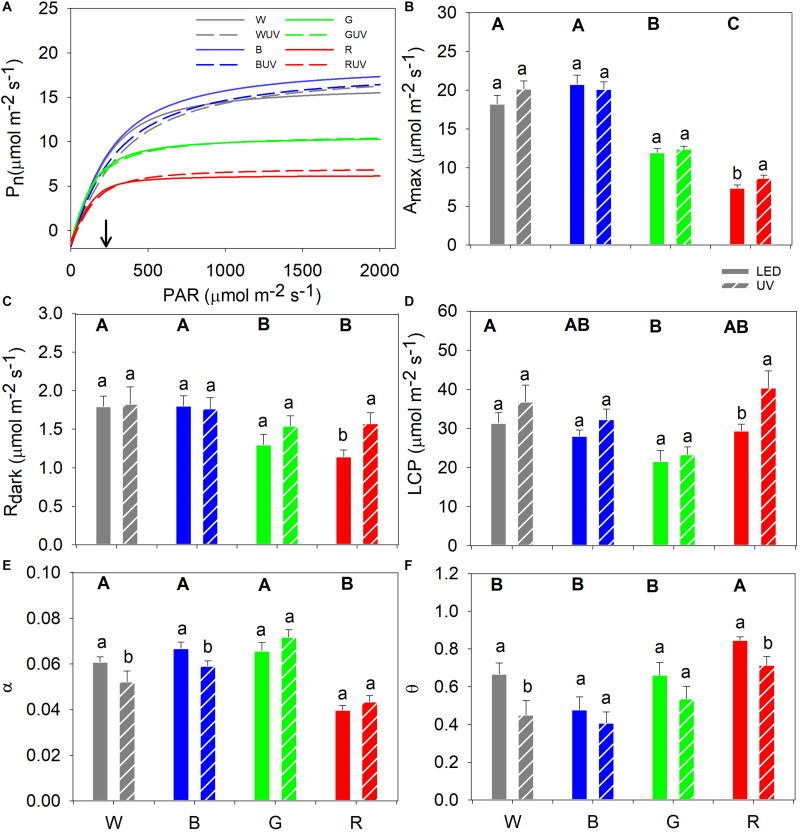
**(A)** Fitted light response curves of cucumber grown under four LED light backgrounds (White, Blue, Green, and Red) without (solid bars) and with (dashed bars) exposure to supplementary UV-B radiation for 14 days [for variation of the data at light saturation refer from panels **(B–F)**], where the arrow indicates the growth PAR. Curve fitted parameters: **(B)** Maximum net assimilation rate (A_max_); **(C)** Dark respiration (R_dark_); **(D)** Light compensation point (LCP); **(E)** Apparent quantum yield of photosynthesis (α); and **(F)** convexity (θ). Bars represent the mean values (*n* = 21 ± SE). Capital letters indicate significant differences between growth light qualities without UV-B and lower-case letters between non-UV-B-exposed plants and UV-B-exposed plants within the same PAR background, both at *P* < 0.05.

Light response curves were measured in the UV-control plants 9–12 days after the start of the UV-B treatment (Figure 3A). For control treatments in the absence of UV-B, plants grown under white and blue light had higher A_max_ followed by the green-light grown plants, whereas the lowest A_max_ was observed in plants grown under red light (Figure 3B). The white- and blue-light-grown plants had higher R_dark_ than those grown under green or red light (Figure 3C). Furthermore, plants grown under white light had the significantly highest LCP and green-light-grown plants the lowest (Figure 3D). The α was significantly lower in plants grown under red light compared to those grown under the other light qualities (Figure 3E). Finally, plants grown under red light had the highest θ while those grown under blue light had the lowest (Figure 3F).

### Monochromatic Light Qualities Induce Different Sensitivity to Saturating Light Conditions

**FIGURE 4:**
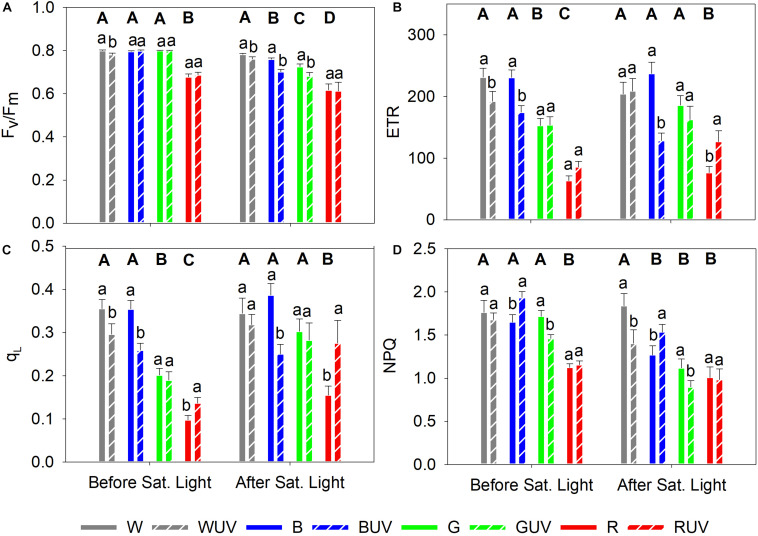
Chlorophyll fluorescence parameters measured in cucumber plants grown under four different PAR qualities (White, Blue, Green, and Red) and without (solid bars) or with (dashed bars) exposure to supplementary UV-B radiation for 14 days, prior to and after a 5 h saturating light treatment (1600 μmol m^–2^ s^–1^). **(A)** Maximum photochemical efficiency (F_v_/F_m_); **(B)** Apparent electron transport rate (ETR); **(C)** Fraction of oxidized PSII (q_L_), **(D)** Non-photochemical quenching (NPQ). Bars represent mean values (Before saturating light: *n* = 21 ± SE; After saturating light *n* = 15 ± SE). Capital letters indicate significant differences between growth light qualities without UV-B and lower-case letters between non-UV-B-exposed plants and UV-B exposed plants within the same PAR background, both at *P* < 0.05.

Chlorophyll fluorescence was measured prior to and immediately after the potentially photoinhibitory saturating light treatment (5 h at a PPFD of 1600 μmol m^–2^ s^–1^) to assess the light stress tolerance of plants grown under different light spectra. Prior to the saturating light treatment, the red-light-grown control plants had significantly lower F_v_/F_m_ compared to plants grown under the other light spectra (Figure 4A). After the saturating light treatment, plants grown under white light had the highest F_v_/F_m_, followed by plants grown under blue, green and red monochromatic light, in descending order (Figure 4A). Prior to the saturating light treatment, the ETR and q_L_ were significantly highest in plants grown in white or blue light, while red light-grown plants had the significantly lowest values (Figures 4B,C). Finally, prior to saturating light application, plants grown under red light had significantly lower NPQ than plants grown under the other spectra, whereas after the saturating light treatment, plants grown under white light were the only ones showing significantly higher NPQ compared with plants grown under the other spectra (Figure 4D).

### Non-destructive Measurements of Chlorophyll and Epidermal Flavonols

**FIGURE 5:**
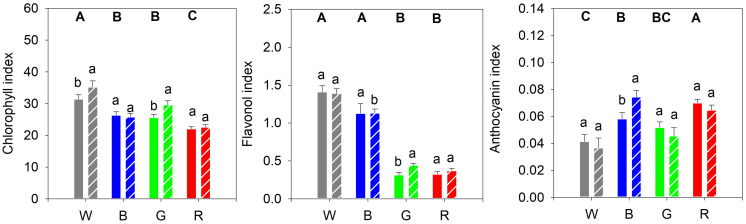
**(A)** Epidermal chlorophyll, **(B)** flavonol and **(C)** anthocyanin content of cucumber plants, as measured with a DUALEX instrument, and grown under different light quality backgrounds (White, Blue, Green, and Red) and without (solid bars) or with (dashed bars) exposure to supplementary UV-B radiation for 14 days. Data are mean values (*n* = 15 ± SE). Capital letters indicate significant differences between growth light qualities without UV-B and lower-case letters between non-UV-B-exposed plants and UV-B exposed plants within the same PAR background, both at *P* < 0.05.

Plants grown under white light had a higher chlorophyll content compared to plants grown under blue or green light. Red light-grown plants exhibited an even lower chlorophyll content (Figure 5A). Cucumber seedlings grown under white and blue light had the highest content of epidermal flavonols, while plants grown under red and green light had the lowest concentrations (Figure 5B). Moreover, plants grown under red light showed the significantly highest anthocyanin content with leaf concentrations decreasing in the following order of light spectra: blue > green > white (Figure 5C).

### UV-B-Induced Effects on Plant Morphology Is Dependent on Monochromatic Light Background

We also analyzed the influence of different monochromatic growth light qualities on UV-induced plant responses. Supplementary UV-B led to a decrease in plant height and INL compared to the corresponding controls (Figures 2A,B, respectively). UV-B also generally reduced plant TDM, except in plants grown under red light (Figure 2C). Moreover, after UV-B exposure, plant TLA decreased significantly in all treatments except for those grown under red light (Figure 2D). Finally, SLM increased in UV-B-treated plants grown in green and red light backgrounds (Figure 2E), whereas leaf number was decreased in all cases (Figure 2F).

### Supplementary UV-B Boosted Photosynthesis in Cucumber Grown Under Red Light

The light response curves of plants grown in the control and UV-B OFTB boxes were compared in order to investigate the effects of supplementary UV-B on photosynthesis parameters in cucumber plants grown under the different spectra (Figure 3A). Only UV-B-exposed plants grown under red light had a statistically significant increase in A_max_, whereas A_max_ was unaltered in all other plants (Figure 3B), which in turn resulted in a similar pattern for the R_dark_ and LCP parameters (Figures 3C,D, respectively). α significantly decreased after UV-B exposure in plants grown under white or blue light, whilst no significant differences were observed in red or green light (Figure 3E). The UV-B exposure of cucumber led to significant decreases in θ in plants grown under white or red light, whereas no significant differences were observed in plants grown under blue or green light (Figure 3F).

### The PAR Spectrum Changes the Effect of UV-B and the Susceptibility to Photoinhibition

To investigate the effect of UV-B on the susceptibility to photoinhibition, plants from all treatments were exposed to 5 h of saturating light after the last day of exposure to UV-B. Prior to the saturating light treatment, only plants grown under white light had a small but significant decrease in F_v_/F_m_ after UV-B exposure (Figure 4A). The F_v_/F_m_ of plants grown under the other light qualities was unaffected by UV-B. After the saturating light treatment, however, plants grown under the red PAR background showed no significant effect of UV-B on F_v_/F_m_, whilst the other PAR backgrounds led to significant decreases in F_v_/F_m_ by UV-B (Figure 4A). Prior to the exposure to saturating light, ETR and q_L_ were reduced after UV-B irradiation in plants grown under white and blue PAR, whereas plants grown under green PAR were unaffected. UV-B exposed plants grown under red PAR were boosted (but non-significantly for ETR) (Figures 4B,C, respectively). After the saturating light treatment, plants that had been exposed to UV-B showed a significant decrease in ETR when grown under blue PAR, while ETR was boosted by UV-B in plants that had red background PAR (Figure 4B). Finally, prior to the saturating light treatment, UV-B increased NPQ in plants grown under blue PAR and decreased in plants grown under green PAR (Figure 4D). After the saturating light treatment, however, UV-B decreased NPQ in white light-grown plants, while NPQ increased in plants grown under blue PAR (Figure 4D).

### UV-B Has a Limited Effect on Pigment Accumulation

After the UV-B treatment, the chlorophyll content increased significantly in plants grown under white and green light compared to their controls (Figure 5A). The strong decrease in epidermal flavonol content seen in plants grown in green or red light, compared with plants grown in white or blue light, was slightly mitigated by UV-B exposure in plants grown in green light (Figure 5B). UV-B exposure of plants grown in blue light led to a significant increase in the anthocyanin content compared to the control, whereas for plants grown under the other light spectra the UV-B did not have any significant effect (Figure 5C).

## Discussion

### Monochromatic Light Differentially Affects Plant Development and Photosynthesis

Development and physiology of cucumber plants grown under white, blue, green or red light differed substantially. Plants grown under white light were more compact, had smaller and thicker leaves compared to plants grown under the other light qualities, yet retained a high biomass accumulation. Notably, plants grown under blue light had a high biomass accumulation and had longer stems and larger leaves. It has been previously shown that blue light effects on growth were dependent on both the plant species studied and the growth conditions. While blue light inhibited stem elongation and leaf expansion, through a reduction in cell expansion (Cosgrove, 1981), other reports instead inferred increased stem elongation and leaf expansion under this light quality (Ahmad and Cashmore, 1997; Hernández and Kubota, 2016). This blue light induction of growth was previously associated to lack of co-action between phytochromes and cryptochromes (Ahmad and Cashmore, 1997; Hernández and Kubota, 2016) and could explain the increased INL and ILA observed in blue-light-grown plants in our study. Plants grown under either broadband white or blue light had similar photosynthetic responses, suggesting that monochromatic blue light was enough to maintain photosynthetic activity. Similar levels of NPQ, ETR, F_q_′/F_m_′ (data not shown) and q_L_ of plants grown under blue or white light support this (Figure 4). Therefore, blue-light-grown plants are able to efficiently use photosynthates for growth.

Our data show that cucumber plants grown under green light had a higher leaf number than plants grown under any of the other light qualities, indicating an increased developmental rate in green PAR. Johkan et al. (2012) showed that small shifts in green wavelengths (510, 520, and 530 nm) had remarkable effects on growth and morphology in red leaf lettuce, and that under a moderate light intensity (300 μmol m^–2^ s^–1^), green light induced a higher biomass accumulation and larger leaf expansion than in plants grown under broadband white light. Green PAR is perceived by both phytochromes and cryptochromes. However, compared with the strong absorption of red and blue wavelengths, respectively, green wavelengths are poorly absorbed by both photoreceptors (Folta and Maruhnich, 2007). Green PAR penetrates deeper into leaf mesophyll than other wavelengths (Smith et al., 2017), increasing absorption of green quanta in light-depleted environments (Sun et al., 1998). The thinner and larger leaves of plants grown under green light could indicate that plants are trying to optimize light absorption by increasing both the light intercepting area and the light transmission to lower levels in the canopy since light scattering in leaves improves light penetration into the leaf especially in the green part of the spectrum (DeLucia et al., 1996). Also, absorption of green light triggered both large and fast biomass accumulation (Figure 2). In fact, green-light-grown plants had slightly larger biomass than plants grown under white or blue light, although A_n_ was lower than in the white- or blue-light-grown plants (Supplementary Figure S1). Thus, plants grown under monochromatic green light managed to sustain growth and development due to improved light absorption at the canopy level as a result of a combination of larger and thinner leaves and improved light transmission. A limitation of this study was that the measurement of photosynthesis was made on the first fully developed leaf (sun leaf) from the top. It shows the acclimation of photosynthesis to the spectrum but does not give a full picture of the canopy photosynthesis. However, it could be expected that the efficient penetration of green light into leaves (DeLucia et al., 1996; Smith et al., 2017) and the distribution of the photosynthetic machinery over large and thin leaves measured under green PAR would allow efficient canopy light absorption and efficient photosynthesis also at the lower leaf levels. If comparing plants grown in white or green light by putting the values of TDM, TLA and A_max_ to 1 for white-light-grown plants, the relative values for the green-light-grown plants will be TDM = 1.07, TLA = 1.65, and A_max_ = 0.65. If these numbers are used to calculate a very crude estimation of the total canopy A_max_, without taking internal shading and light acclimation into consideration, the white-light-grown plants will have canopy A_max_ of 1 × 1 = 1, while the green-light-grown plants will have 1.65 × 0.65 = 1.07. This crude relative photosynthesis rate at light saturation on canopy level actually fits to the relative TDM for the green-light-grown plants.

In nature, a green-light-enriched environment is an indication of overgrowing vegetation triggering a shade-avoidance response resulting in stem elongation and upward leaf orientation (Zhang et al., 2011; Zhang and Folta, 2012; Wang et al., 2015). Moreover, Folta (2004) suggested that supplementary green light irradiation induces early hypocotyl elongation. Inhibition of stem elongation is a phytochrome-dependent response, and the wavelengths of our green growth light fall precisely outside the range of the phytochrome action spectrum, thus simulating a light environment lacking the red wavelengths. In our study, green light grown plants were significantly taller than the plants grown under red and broadband white light. This suggests that plants grown under green light, in addition to having thinner and larger leaves, also tried to optimize light absorption by growing taller in response to a red-depleted light environment.

While blue growth light did not change any of the plants’ photosynthetic parameters compared with plants grown in white light, plants grown under green light showed decreased A_max_, R_dark_, and LCP, while maintaining α and θ at the same levels as plants grown in white or blue light (Figure 3). All these changes correspond to low-light acclimation of photosynthesis (Givnish, 1988), accompanied by lower ETR and q_L_, and maintained NPQ, resulting in an NPQ increase in proportion to ETR. Plants grown under green light showed a large decrease in the light saturated A_max_ compared with plants grown in white light. However, at the lower growth irradiance (210 μmol m^–2^ s^–1^) the decrease of A_n_ was considerably smaller. Since the total biomass production was even higher in plants grown under green light than in plants grown under white PAR, this suggests that the green-light-grown-plants were not source limited.

Generally, plants grown under monochromatic red PAR cannot sustain normal photosynthetic activity (Hogewoning et al., 2010; Trouwborst et al., 2010). Red-light-grown plants had a severely decreased A_max_ so that the growth was source limited with a much decreased A_n_ and a lower biomass production. For plants grown under red light, A_max_ decreased more than in plants grown under green light, and when R_dark_ decreased, so did α, leaving LCP unaffected. Plants grown under red PAR were the only ones showing a lower α, which deviates from the normal pattern of acclimation to low light level (Givnish, 1988). It could be expected a lower α during stomatal limitation of A_n_, but this was not the case since C_i_ was unaffected (Supplementary Figure S1), suggesting instead a strong biochemical limitation. A decrease in α could also be an indication of photoinhibition (Ögren and Sjöström, 1990). Indeed, F_v_/F_m_ was significantly lower in plants grown under red PAR than in those grown under white light which agrees with previous studies showing dysfunctional photosynthesis in cucumber grown in the absence of blue PAR during growth (Hogewoning et al., 2010). NPQ is a protective mechanism through which plants dissipate excessive energy in the form of heat (Maxwell and Johnson, 2000). The low NPQ of red-light-grown cucumber suggests a low heat dissipation, which could be associated with a strong down-regulation of the photosynthetic process. Furthermore, impairment of photosynthesis could explain the growth inhibition observed in cucumber grown under red light, manifested as plants with the smallest stem Ø and lowest biomass accumulation (LDM, SDM, and TDM) compared with plants grown under the other light qualities. Red-light-grown cucumber also had highest LMR, suggesting that the plants allocated as many resources as possible toward leaves to mitigate growth inhibition.

To evaluate how light acclimation affected general light stress tolerance, plants were subjected to a 5-h photoinhibitory treatment. The applied high light stress decreased F_v_/F_m_ in all treatments. However, white-light-grown plants had the smallest decrease, suggesting a better ability to cope with saturating light conditions. Plants grown under blue, green or red light showed gradually lower F_v_/F_m_, indicating increasing sensitivity toward saturating light in that order. ETR and q_L_ also gradually decreased in the same order, although it was only plants grown under red light that showed a statistically significant change (Supplementary Table S3). NPQ decreased in plants grown under all different monochromatic light qualities, indicating that energy dissipation due to down-regulation of PSII increased. This was manifested as a lower F_v_/F_m_, at the expense of light-regulated heat dissipation (NPQ), particularly in red-light-grown plants.

### Metabolite Composition Is Affected by Spectral Composition

We show a relation between the spectral composition and accumulation of secondary metabolites. The non-destructive measurements showed that plants grown under white light had the highest total chlorophyll content, followed by plants grown under blue, green and red light in decreasing order. This indicates the importance of blue light in the light environment for chlorophyll formation in cucumber during growth. The effects of broadband white on flavonoid content differs between plant species, but in our study, the cucumber plants grown under white PAR had the highest leaf epidermal flavonol content followed by plants grown under blue light. Plants grown under green or red light had significantly lower leaf epidermal flavonol content. In fact, Ouzounis et al. (2014) showed an increase in flavonoid content of roses, campanulas and chrysanthemums with an increasing proportion of blue light in a red background light, in contrast to a low flavonoid content in plants grown under monochromatic red light.

Anthocyanins often function as photoprotective pigments, reducing the amount of light that penetrates the leaf epidermis and preventing damage caused by excessive incident light (Day et al., 1993). In our study, red-light-grown plants had the highest anthocyanin content, followed by plants grown in blue, green and white light, respectively. This suggests that plants grown under red PAR induced anthocyanin accumulation in the leaf epidermis in order to reduce incident light and protect the photosynthetic system from further damage. An increased anthocyanin accumulation in plants grown under red light has previously been reported in red cabbage (Mizuno et al., 2011), bilberries (Zoratti et al., 2014), and lettuce (Garrett Owen and Lopez, 2015). The accumulation of anthocyanins, as well as the decreased F_v_/F_m_ and α suggest that the red light is a stress factor in cucumber, but that the accumulated anthocyanins were insufficient to protect the leaves from light stress by the red growth light.

### Monochromatic Light Treatments Modify UV-B Responses in Cucumber

We found that a lower than ambient level of supplementary UV-B exposure led to decreased extension growth (height and INL) that mostly affected plants grown in green and red PAR, in which SLM increased. This indicates that plants developed shorter stems and thicker leaves to acclimate to UV-B (Jenkins, 2017). Moreover, a clear partitioning of biomass from stem to leaves were observed in plants grown under all light qualities after exposure to UV-B, manifested as a higher LMR. The height, leaf number and TDM were reduced and the effects were smallest in plants grown under red light. This either suggests that red-light-grown plants are less sensitive to the low UV-B level used with regards to morphology, or that growth inhibition caused by red growth light itself overrides the effects of supplementary UV-B. It should also be noted that the effects of UV-B are dose dependent and high doses of UV-B radiation, much higher than those used in this study, can cause distress and reduce plant growth and development (Hideg et al., 2013). Low level supplementary UV-B exposures reduced α in plants grown in white light although A_max_ remained unaltered. In addition, supplementary UV-B led to decreased F_v_/F_m_, ETR, and q_L_, while no effects on NPQ were observed in white-light-grown plants, suggesting a slight down-regulation of photosynthesis compared with the corresponding control plants. UV-B radiation can decrease photosynthetic capacity through a number of high dose mechanisms targeting for instance both the donor and the acceptor sides of Photosystem II or Rubisco (Jordan et al., 2016). Other such mechanisms include photodegradation of light-absorbing pigments (Prasad et al., 2004), such as chlorophyll (Strid and Porra, 1992; Nedunchezhian and Kulandaivelu, 1997) and carotenoids. However, we show that the chlorophyll content increases in cucumber grown in white light treated with UV-B compared with the corresponding control, contributing to the contradicting conclusions described in literature (Jordan et al., 2016). This may be due to different levels of UV-B used in in different studies, as well as the use of different UV-B to PAR ratios.

The use of blue light may prevent damage caused by high UV-B levels (Hoffmann et al., 2015; Escobar-Bravo et al., 2017). Hoffmann et al. (2015) demonstrated that high intensities of blue light (300 μmol m^–2^ s^–1^) improved the photosynthetic performance of pepper plants exposed to UV-B. The reduced UV-B damage could be explained by reduced degradation of photosynthetic pigments and by increased accumulation of epidermal UV-absorbing flavonoids synergistically induced by blue light and UV-B (Nascimento et al., 2013; Ouzounis et al., 2014; Hoffmann et al., 2015). However, this was not observed in our study using low level UV-B. Together with an unchanged A_max_, decreased ETR and q_L_, as well as an increased NPQ in plants grown under blue PAR, we show that monochromatic blue light does not improve plant acclimation or increase photoprotection to UV-B. The production of anthocyanins has previously been shown to increase by a combination of blue PAR and UV-A in turnip seedlings (Wang et al., 2012) and apple (Arakawa et al., 1985). This agrees with our results.

Cucumbers appear less susceptible to low levels of UV-B when grown in green light, since a decrease in NPQ was the only significant UV-B-induced change in the photosynthesis parameters, suggesting a slightly increased energy flow to photochemistry. This was accompanied by higher concentrations of chlorophyll and flavonols. Most interestingly, adding UV-B to red PAR growth light boosted photosynthesis of cucumber plants compared with the corresponding red PAR control. Although a higher A_max_, LCP, and R_dark_ indicated increased photosynthesis, this was not due to improved photochemistry (no increase in α), but rather due to a positive effect on the biochemical processes regulating CO_2_ assimilation. Moreover, supplementary UV-B had no negative effect on F_v_/F_m_ and did not induce any additional stress to the photosynthetic machinery of red-light-grown plants. Additionally, no changes in epidermal pigment content (chlorophyll, flavonol, and anthocyanin) were observed when supplementary UV-B was added to a red PAR growth light. The positive effects on photosynthesis may explain the lack of growth inhibition caused by UV-B in a background of red growth light.

### Light Spectra and UV-B Affects the Susceptibility to Photoinhibition

The use of realistic levels of UV-B radiation play an important role in enhancing photoprotection under saturating light (Wargent et al., 2015), rather than causing further damage to the photosynthetic apparatus. After photoinhibition, plants grown under all different light qualities (but without UV-B) showed a lowered F_v_/F_m_ along a distinct gradient with the smallest effect in plants grown in white light, via blue- and green-light-grown plants, to the largest effect in plants grown in red light. In addition, supplementary UV-B lowered F_v_/F_m_ even further in plants grown in each different light quality, except for red-light-grown plants, where F_v_/F_m_ was unaffected. This is of particular interest since ETR and q_L_ decreased after 2 weeks of UV-B exposure prior to photoinhibitory treatment in blue-light-grown plants (unaffected A_max_; Figure 3), remained the same in green-light-grown plants (slightly reduced A_max_), but increased in red-light-grown cucumber (strongly reduced A_max_; lower F_v_/F_m_). We suggest that the low UV-B levels used in our study create eustress to activate defense systems, e.g., antioxidants, which put the plants in a state of “low alert” toward other stresses that may involve oxidative stress (Hideg et al., 2013), including photoinhibition. Obviously, addition of UV-B is not enough to fully overcome stress induced by red light given in the growth phase, but both A_max_ and q_L_ are significantly higher in UV-B-treated photoinhibited plants than in plants that had been grown solely in red light before photoinhibition (with a clear trend also in ETR). In fact, absolute levels of q_L_ and ETR in UV-B-treated photoinhibited red-light-grown plants were similar as in UV-B-treated photoinhibited green- or blue-light-grown plants. If the UV-B treatment induces a low-level alert against other stresses (Jansen et al., 2019), it seems that such another stress (in this case photoinhibition) has to be of a certain magnitude for a plant to benefit from the UV-B treatment. Thus, the effect of UV-B mitigating a second stress, such as photoinhibition, follows a gradient from no beneficial effect at all in non-stressed leaves to a beneficial effect in already light stressed leaves.

## Conclusion

In agreement with our first hypothesis, we show that different monochromatic light backgrounds exert different responses in growth and physiology in cucumber. Monochromatic green and blue growth light, but not red, enabled normal photosynthetic functioning of leaves of cucumber plants without compromising biomass accumulation. Despite being exposed to the same light level, plants grown in green light showed low light acclimation of photosynthesis, but because of the changed canopy architecture with larger and thinner leaves these plants had the highest total biomass production. On the other hand, in plants grown in red light, the low light acclimation was more pronounced and accompanied by light stress symptoms that reduced F_v_/F_m_ and also led to reduced growth.

Our data confirmed our second hypothesis that cucumber responses to UV-B are highly dependent of the spectrum of monochromatic growth light. Supplementary UV-B radiation decreased plant growth and development in plants grown under blue, green and white but not under red light. Although the results suggest dysfunctional photosynthesis in plants grown under red light, UV-B boosted some photosynthetic parameters, actually increasing the potential carbon gain. Thus, UV-B and red light could act synergistically on priming the plant antioxidant capacity and diminish negative effects of photoinhibition. However, a more in-depth study of the metabolic and molecular pathways and antioxidants triggered by the treatments is required to fully explain our findings.

The findings presented here could have a positive impact on horticultural settings. By using the right monochromatic light in early stages of cucumber production, plant development may be accelerated and thus decreasing overall production time.

## Data Availability Statement

The original contributions presented in the study are included in the article/Supplementary Material. Further inquiries can be directed to the corresponding author/s.

## Author Contributions

CP performed the experiment and most of the analysis, and prepared the manuscript. VC-A commented on the manuscript. LM assisted with analysis and worked on the manuscript. ER helped analyzing data and edited the manuscript. C-OO worked with editing of the manuscript. ÅS prepared spectral figures and edited the manuscript. All authors contributed to the article and approved the submitted version.

## Conflict of Interest

The authors declare that the research was conducted in the absence of any commercial or financial relationships that could be construed as a potential conflict of interest.

## Abbreviations

α: apparent quantum yield of photosynthesis
θ: curvature
A_n_: net photosynthetic rate
A_max_: maximum net assimilation rate
C_i_: intracellular CO_2_ concentration
CO_2_: carbon dioxide
DM%: Dry mass percentage
DM: Dry mass
E: transpiration rate
ETR: electron transport rate
FM: fresh mass
F_v_/F_m_: maximum photochemical efficiency of PSII
F_q_ ′ /F_m_ ′: quantum yield or operation efficiency of PSII
g_s_: stomatal conductance
ILA: individual leaf area
INL: internode length
LCP: light compensation point
LMR: leaf mass ratio
NPQ: non-photochemical quenching
PAR: photosynthetically active radiation
q_L_: fraction of open PSII centres
PSII: photosystem II
R_dark_: dark respiration
ROS: reactive oxygen species
SLM: specific leaf mass
TLA: total leaf area
UV-B: Ultraviolet B
Ø: stem diameter.
