# Supplementary material for: Spectral Composition of Light Affects Sensitivity to UV-B and Photoinhibition in Cucumber
Source: Front Plant Sci. 2021 Jan 5;11:610011. doi: 10.3389/fpls.2020.610011 (PMC7813804; doi:10.3389/fpls.2020.610011)
Supplement: Supplementary file 1 [file Table_1.DOCX]

**Supplementary Table S1.** Biomass accumulation of cucumber plants grown under four different light backgrounds prior to UV-B exposure. Data are mean values (n = 5 ± SE). Letters indicate significant difference between different PAR backgrounds at P-value < 0.05 within rows.

| Parameter | Treatment | | | |
| --- | --- | --- | --- | --- |
|  | **Day 0** | | | |
|  | **White** | **Blue** | **Green** | **Red** |
| *Height (cm)* | 4.1±0.1**^d^** | 6.5±0.3**^a^** | 5.1±0.1**^b^** | 4.5±0.1**^c^** |
| *Stem Ø (mm)* | 2.8±0.1**^b^** | 2.9±0.1**^b^** | 3.1±0.1**^a^** | 2.6±0.1**^c^** |
| *LDM (g)* | 0.20±0.01**^a^** | 0.16±0.05**^b^** | 0.20±0.01**^a^** | 0.16±0.01**^b^** |
| *SDM (g)* | 0.018±0.001**^c^** | 0.025±0.011**^a^** | 0.025±0.001**^a^** | 0.020±0.006**^b^** |
| *TDM (g)* | 0.22±0.02**^a^** | 0.18±0.01**^b^** | 0.22±0.01**^a^** | 0.18±0.01**^b^** |
| *DM%* | 10.1±0.1**^b^** | 11.6±0.1**^ab^** | 11.6±0.1**^ab^** | 12.1±0.2**^a^** |
| *LMR* | 0.914±0.003**^a^** | 0.866±0.004**^c^** | 0.884±0.004**^b^** | 0.886±0.003**^b^** |
| *TLA (cm^2^)* | 67±5**^c^** | 54±5**^d^** | 103±6**^a^** | 86±5**^b^** |
| *SLM (g cm^-2^)* | 0.0291±0.0013**^a^** | 0.0275±0.0004**^a^** | 0.0195±0.0005**^b^** | 0.0200±0.0008**^b^** |
| *Leaf no.* | 1.5±0.1**^b^** | 1.3±0.1**^b^** | 2±0**^a^** | 1.6±0.1**^b^** |
