# Supplementary material for: Spectral Composition of Light Affects Sensitivity to UV-B and Photoinhibition in Cucumber
Source: Front Plant Sci. 2021 Jan 5;11:610011. doi: 10.3389/fpls.2020.610011 (PMC7813804; doi:10.3389/fpls.2020.610011)
Supplement: Supplementary file 2 [file Table_2.DOCX]

**Supplementary Table S2.**  Epidermal chlorophyll, flavonoid and anthocyanin content of cucumber plants grown under different light quality backgrounds (White, Blue, Green and Red), measured using a DUALEX instrument, prior to UV-B exposure. Data are mean values (n = 5 ± SE). Letters indicate significant difference between different PAR backgrounds at P < 0.05 within rows.

| Parameter | Treatment | | | |
| --- | --- | --- | --- | --- |
|  | **Day 0** | | | |
|  | **White** | **Blue** | **Green** | **Red** |
| *Chlorophyll* | 24.7±0.3**^a^** | 22.3±0.3**^bc^** | 23.4±0.6**^ab^** | 21.1±0.6**^c^** |
| *Flavonoid* | 1.16±0.03**^a^** | 1.17±0.02**^a^** | 0.52±0.01**^b^** | 0.35±0.01**^c^** |
| *Anthocyanin* | 0.065±0.001**^c^** | 0.080±0.002**^a^** | 0.059±0.003**^c^** | 0.072±0.002**^b^** |
