# Supplementary material for: Spectral Composition of Light Affects Sensitivity to UV-B and Photoinhibition in Cucumber
Source: Front Plant Sci. 2021 Jan 5;11:610011. doi: 10.3389/fpls.2020.610011 (PMC7813804; doi:10.3389/fpls.2020.610011)
Supplement: Supplementary file 3 [file Table_3.DOCX]

**Supplementary Table S3**. Summary of the ANOVA models used to estimate significant main effects of growing light backgrounds on plant biomass (destructive harvest), photosynthesis, chlorophyll fluorescence and epidermal pigment content measured with DUALEX .

df_num_ (numerator degrees of freedom), df_den_ (denominator degrees of freedom)

| **Destructive Harvest Day 0 of UV treatment – Light backgrounds** |  |  |  |  |
| --- | --- | --- | --- | --- |
| **Source** | **df_num_** | **df_den_** | ***F*** | ***P*** |
| **Height** |  |  |  |  |
| Treatment | 3 | 114 | 63.720 | **<**0.0001 |
|  |  |  |  |  |
| **INL** |  |  |  |  |
| Treatment | 3 | 112 | 39.5785 | <0.0001 |
|  |  |  |  |  |
| **Stem Ø** |  |  |  |  |
| Treatment | 3 | 114 | 8.9357 | <0.0001 |
|  |  |  |  |  |
| **LFM** |  |  |  |  |
| Treatment | 3 | 114 | 15.8176 | <0.0001 |
|  |  |  |  |  |
| **SFM** |  |  |  |  |
| Treatment | 3 | 114 | 60.9423 | <0.0001 |
|  |  |  |  |  |
| **TFM** |  |  |  |  |
| Treatment | 3 | 114 | 13.2522 | <0.0001 |
|  |  |  |  |  |
| **LDM** |  |  |  |  |
| Treatment | 3 | 114 | 15.7186 | <0.0001 |
|  |  |  |  |  |
| **SDM** |  |  |  |  |
| Treatment | 3 | 114 | 22.0172 | <0.0001 |
|  |  |  |  |  |
| **TDM** |  |  |  |  |
| Treatment | 3 | 114 | 13.5508 | <0.0001 |
|  |  |  |  |  |
| **Leaf no** |  |  |  |  |
| Treatment | 3 | 114 | 19.172 | <0.0001 |
|  |  |  |  |  |
| **TLA** |  |  |  |  |
| Treatment | 3 | 112 | 38.5710 | <0.0001 |
|  |  |  |  |  |
| **ILA** |  |  |  |  |
| Treatment | 3 | 112 | 8.0496 | 0.0001 |
|  |  |  |  |  |
| **DM%** |  |  |  |  |
| Treatment | 3 | 114 | 76.64 | <0.0001 |
|  |  |  |  |  |
| **SLM** |  |  |  |  |
| Treatment | 3 | 113 | 65.224 | <0.0001 |
|  |  |  |  |  |
| **LMR** |  |  |  |  |
| Treatment | 3 | 114 | 51.922 | <0.0001 |
|  |  |  |  |  |
| **Destructive Harvest Day 14 of UV treatment – Light backgrounds** |  |  |  |  |
| **Source** | **df_num_** | **df_den_** | ***F*** | ***P*** |
| **Height** |  |  |  |  |
| Treatment | 3 | 162 | 77.5023 | <0.0001 |
|  |  |  |  |  |
| **INL** |  |  |  |  |
| Treatment | 3 | 162 | 74.5327 | <0.0001 |
|  |  |  |  |  |
| **Stem Ø** |  |  |  |  |
| Treatment | 3 | 162 | 31.3422 | <0.0001 |
|  |  |  |  |  |
| **LFM** |  |  |  |  |
| Treatment | 3 | 162 | 54.9872 | <0.0001 |
|  |  |  |  |  |
| **SFM** |  |  |  |  |
| Treatment | 3 | 162 | 50.9848 | <0.0001 |
|  |  |  |  |  |
| **TFM** |  |  |  |  |
| Treatment | 3 | 162 | 47.9577 | <0.0001 |
|  |  |  |  |  |
| **LDM** |  |  |  |  |
| Treatment | 3 | 162 | 56.4431 | <0.0001 |
|  |  |  |  |  |
| **SDM** |  |  |  |  |
| Treatment | 3 | 162 | 60.1421 | <0.0001 |
|  |  |  |  |  |
| **TDM** |  |  |  |  |
| Treatment | 3 | 162 | 55.2584 | <0.0001 |
|  |  |  |  |  |
| **Leaf no** |  |  |  |  |
| Treatment | 3 | 162 | 40.3850 | <0.0001 |
|  |  |  |  |  |
| **TLA** |  |  |  |  |
| Treatment | 3 | 162 | 42.4296 | <0.0001 |
|  |  |  |  |  |
| **ILA** |  |  |  |  |
| Treatment | 3 | 162 | 19.9036 | <0.0001 |
|  |  |  |  |  |
| **DM%** |  |  |  |  |
| Treatment | 3 | 162 | 32.3819 | <0.0001 |
|  |  |  |  |  |
| **SLM** |  |  |  |  |
| Treatment | 3 | 162 | 79.253 | <0.0001 |
|  |  |  |  |  |
| **LMR** |  |  |  |  |
| Treatment | 3 | 162 | 50.591 | <0.0001 |
|  |  |  |  |  |

| **Destructive Harvest Day 0 of UV treatment – Contrast Light Backgrounds** | | | | | | |
| --- | --- | --- | --- | --- | --- | --- |
|  | | | | | | |
|  | **White × Blue** | **White × Green** | **White × Red** | **Blue × Green** | **Blue × Red** | **Red × Green** |
| **Height** | 0 | 1.738e^-12^ | 4.668e^-05^ | 1.686e^-8^ | 1.110e^-15^ | 6.911e^-05^ |
|  |  |  |  |  |  |  |
| **INL** | 2.042e^-14^ | 0.2311 | 0.6796 | 0 | 0 | 0.6796 |
|  |  |  |  |  |  |  |
| **Stem Ø** | 0.2354 | 0.0099 | 0.2354 | 0.2354 | 0.0030 | 0 |
|  |  |  |  |  |  |  |
| **LFM** | 0.0025 | 0.0031 | 0.4331 | 0.00143 | 2.598e^-9^ | 3.264e^-04^ |
|  |  |  |  |  |  |  |
| **SFM** | 6.661e^-15^ | 0 | 2.020e^-10^ | 0.0500 | 0.0245 | 5.983e^-05^ |
|  |  |  |  |  |  |  |
| **TFM** | 1.0000 | 9.144e^-07^ | 1.0000 | 9.144e^-07^ | 1.0000 | 3.509e^-05^ |
|  |  |  |  |  |  |  |
| **LDM** | 1.389e^-04^ | 0.4571 | 2.472e^-04^ | 1.3887e^-06^ | 0.8194 | 3.1328e^-06^ |
|  |  |  |  |  |  |  |
| **SDM** | 3.089e^-07^ | 3.270e^-10^ | 0.0026 | 0.1165 | 0.0300 | 4.1761e^-04^ |
|  |  |  |  |  |  |  |
| **TDM** | 0.0042 | 0.1150 | 0.0027 | 4.893e^-06^ | 0.8232 | 2.227e^-06^ |
|  |  |  |  |  |  |  |
| **Leaf no** | 0.3304 | 9.265e^-06^ | 0.2861 | 8.416e^-04^ | 0.1703 | 9.047e^-06^ |
|  |  |  |  |  |  |  |
| **TLA** | 0.0389 | 2.272e^-11^ | 3.580e^-05^ | 0.0000 | 4.873e^-09^ | 0.0034 |
|  |  |  |  |  |  |  |
| **ILA** | 1.0000 | 0.0064 | 0.0046 | 0.0025 | 0.0015 | 1.0000 |
|  |  |  |  |  |  |  |
| **DM%** | 0 | 0 | 0 | 0.8247 | 0.0710 | 0.0628 |
|  |  |  |  |  |  |  |
| **SLM** | 0.2543 | 0 | 0 | 2.438e^-13^ | 0 | 0.2542 |
|  |  |  |  |  |  |  |
| **LMR** | 0 | 6.870e^-12^ | 1.082e^-11^ | 0.0015 | 0.0014 | 0.8816 |
| **Destructive Harvest Day 14 of UV treatment - Contrast Light Backgrounds** | | | | | | |
|  | | | | | | |
|  | **White × Blue** | **White × Green** | **White × Red** | **Blue × Green** | **Blue × Red** | **Red × Green** |
| **Height** | 0 | 0 | 3.862e^-05^ | 0.7240 | 2.487e^-14^ | 8.526e^-14^ |
|  |  |  |  |  |  |  |
| **INL** | 0 | 1.693e^-11^ | 0.0059 | 1.954e^-14^ | 0 | 5.715e^-06^ |
|  |  |  |  |  |  |  |
| **Stem Ø** | 1.000 | 1.000 | 3.399e^-12^ | 0.9991 | 3.041e^-11^ | 1.665e^-13^ |
|  |  |  |  |  |  |  |
| **LFM** | 4.355e^-04^ | 2.328e^-08^ | 2.505e^-08^ | 0 | 0.0233 | 0 |
|  |  |  |  |  |  |  |
| **SFM** | 2.119e^-11^ | 1.563e^-12^ | 0.0432 | 0.527 | 1.110e^-15^ | 0 |
|  |  |  |  |  |  |  |
| **TFM** | 0.0165 | 6.285e^-11^ | 2.421e^-05^ | 6.043e^-06^ | 4.995e^-10^ | 0 |
|  |  |  |  |  |  |  |
| **LDM** | 4.826e^-05^ | 0.0621 | 0 | 1.579e^-08^ | 3.527e^-07^ | 0 |
|  |  |  |  |  |  |  |
| **SDM** | 1.001e^-06^ | 6.065e^-06^ | 4.211e^-08^ | 0.5946 | 0 | 0 |
|  |  |  |  |  |  |  |
| **TDM** | 0.2051 | 0.0257 | 2.220e^-15^ | 6.496e^-04^ | 1.993e^-12^ | 0 |
|  |  |  |  |  |  |  |
| **Leaf no** | 0.0381 | 1.265e^-13^ | 0.3554 | 0 | 0.0036 | 2.038e^-11^ |
|  |  |  |  |  |  |  |
| **TLA** | 0.0031 | 0 | 0.0259 | 1.672e^-11^ | 0.4073 | 1.687e^-13^ |
|  |  |  |  |  |  |  |
| **ILA** | 1.445e^-08^ | 4.892e^-09^ | 0.0212 | 0.8092 | 8.836e^-04^ | 4.793e^-04^ |
|  |  |  |  |  |  |  |
| **DM%** | 5.682e^-12^ | 0 | 5.151e^-13^ | 0.1535 | 0.6241 | 0.2884 |
|  |  |  |  |  |  |  |
| **SLM** | 0 | 0 | 0 | 2.569e^-06^ | 0.0045 | 0.0432 |
|  |  |  |  |  |  |  |
| **LMR** | 0 | 5.4318e^-06^ | 0.0065 | 4.438e^-07^ | 0 | 2.886e^-11^ |

| **Gas exchange – Light backgrounds** |  |  |  |  |
| --- | --- | --- | --- | --- |
| **Source** | **df_num_** | **df_den_** | ***F*** | ***P*** |
| **R_dark_** |  |  |  |  |
| Treatment | 3 | 78 | 8.12132 | 0.0001 |
|  |  |  |  |  |
| **α** |  |  |  |  |
| Treatment | 3 | 78 | 36.9156 | <0.0001 |
|  |  |  |  |  |
| **LCP** |  |  |  |  |
| Treatment | 3 | 78 | 3.2657 | 0.0257 |
|  |  |  |  |  |
| **A_max_** |  |  |  |  |
| Treatment | 3 | 78 | 76.1241 | <0.0001 |
|  |  |  |  |  |
| **θ** |  |  |  |  |
| Treatment | 3 | 78 | 7.985 | 0.0001 |
|  |  |  |  |  |

| **Gas exchange - Light backgrounds contrasts** | | | | | | |
| --- | --- | --- | --- | --- | --- | --- |
|  | | | | | | |
|  | **White × Blue** | **White × Green** | **White × Red** | **Blue × Green** | **Blue × Red** | **Red × Green** |
| **R_dark_** | 0.9613 | 0.0231 | 0.0010 | 0.0231 | 0.0010 | 0.5300 |
|  |  |  |  |  |  |  |
| **α** | 0.5818 | 0.5818 | 8.559e^-09^ | 0.8433 | 9.059e^-11^ | 1.579e^-10^ |
|  |  |  |  |  |  |  |
| **LCP** | 0.7455 | 0.0180 | 0.9451 | 0.2175 | 0.9451 | 0.0990 |
|  |  |  |  |  |  |  |
| **A_max_** | 0.0959 | 1.296e^-06^ | 1.110e^-15^ | 8.674e^-09^ | 0 | 8.674e^-09^ |
|  |  |  |  |  |  |  |
| **θ** | 0.2410 | 0.9337 | 0.0116 | 0.2410 | 0.0025 | 0.0116 |

| **CF prior to photoinhibitory treatment – Light backgrounds** |  |  |  |  |
| --- | --- | --- | --- | --- |
| **Source** | **df_num_** | **df_den_** | ***F*** | ***P*** |
| ***F_v_/F_m_*** |  |  |  |  |
| Treatment | 3 | 78 | 27.702 | **<**0.0001 |
|  |  |  |  |  |
| **ETR** |  |  |  |  |
| Treatment | 3 | 78 | 61.9777 | **<**0.0001 |
|  |  |  |  |  |
| **NPQ** |  |  |  |  |
| Treatment | 3 | 78 | 20.9235 | **<**0.0001 |
|  |  |  |  |  |
| **q_L_** |  |  |  |  |
| Treatment | 3 | 78 | 69.5988 | **<**0.0001 |

| **CF after photoinhibitory treatment – Light backgrounds** |  |  |  |  |
| --- | --- | --- | --- | --- |
| **Source** | **df_num_** | **df_den_** | ***F*** | ***P*** |
| ***F_v_/F_m_*** |  |  |  |  |
| Treatment | 3 | 52 | 21.35 | **<**0.0001 |
|  |  |  |  |  |
| **ETR** |  |  |  |  |
| Treatment | 3 | 52 | 29.9450 | **<**0.0001 |
|  |  |  |  |  |
| **NPQ** |  |  |  |  |
| Treatment | 3 | 52 | 8.7975 | 0.0001 |
|  |  |  |  |  |
| **q_L_** |  |  |  |  |
| Treatment | 3 | 52 | 16.4188 | **<**0.0001 |

| **CF prior to photoinhibitory treatment- Light backgrounds contrasts** | | | | | | |
| --- | --- | --- | --- | --- | --- | --- |
|  | | | | | | |
|  | **White × Blue** | **White × Green** | **White × Red** | **Blue × Green** | **Blue × Red** | **Red × Green** |
| ***F_v_/F_m_*** | 0.4923 | 0.8879 | 4.449e^-13^ | 0.8879 | 1.421e^-12^ | 7.438e^-13^ |
|  |  |  |  |  |  |  |
| **ETR** | 0.9813 | 8.582e^-05^ | 0 | 8.582e^-05^ | 0 | 3.317e^-08^ |
|  |  |  |  |  |  |  |
| **NPQ** | 1 | 1 | 4.208e^-06^ | 1 | 7.135e^-06^ | 4.208e^-06^ |
|  |  |  |  |  |  |  |
| **q_L_** | 0.9742 | 2.842e^-07^ | 0 | 2.842e^-07^ | 0 | 3.780e^-07^ |
|  |  |  |  |  |  |  |
| **CF after photoinhibitory treatment- Light backgrounds contrasts** | | | | | | |
|  | | | | | | |
|  | **White × Blue** | **White × Green** | **White × Red** | **Blue × Green** | **Blue × Red** | **Red × Green** |
| ***F_v_/F_m_*** | 6.068e^-03^ | 1.097e^-05^ | 9.556e^-06^ | 6.068e^-03^ | 1.168e^-05^ | 3.846e^-03^ |
|  |  |  |  |  |  |  |
| **ETR** | 0.4121 | 0.4408 | 1.481e^-07^ | 0.1457 | 5.419e^-09^ | 2.727e^-06^ |
|  |  |  |  |  |  |  |
| **NPQ** | 0.0093 | 0.0007 | 5.849e^-05^ | 0.7373 | 0.3297 | 0.7373 |
|  |  |  |  |  |  |  |
| **q_L_** | 0.6585 | 0.6585 | 2.094e^-05^ | 0.1890 | 1.484e^-06^ | 0.0006 |

| **DUALEX Day 0 of UV treatment – Light backgrounds** |  |  |  |  |
| --- | --- | --- | --- | --- |
| **Source** | **df_num_** | **df_den_** | ***F*** | ***P*** |
| **Chl** |  |  |  |  |
| Treatment | 3 | 99 | 12.5973 | <0.0001 |
|  |  |  |  |  |
| **Flav** |  |  |  |  |
| Treatment | 3 | 99 | 386.4103 | <0.0001 |
|  |  |  |  |  |
| **Anth** |  |  |  |  |
| Treatment | 3 | 99 | 16.9934 | <0.0001 |

| **DUALEX Day 14 of UV treatment – Light backgrounds** |  |  |  |  |
| --- | --- | --- | --- | --- |
| **Source** | **df_num_** | **df_den_** | ***F*** | ***P*** |
| **Chl** |  |  |  |  |
| Treatment | 3 | 42 | 14.4399 | <0.0001 |
|  |  |  |  |  |
| **Flav** |  |  |  |  |
| Treatment | 3 | 42 | 54.4536 | <0.0001 |
|  |  |  |  |  |
| **Anth** |  |  |  |  |
| Treatment | 3 | 42 | 10.5052 | <0.0001 |

| **DUALEX Day 0 of UV treatment – Light backgrounds contrasts** | | | | | | |
| --- | --- | --- | --- | --- | --- | --- |
|  | | | | | | |
|  | **White × Blue** | **White × Green** | **White × Red** | **Blue × Green** | **Blue × Red** | **Red × Green** |
| **Chl** | 3.892e^-04^ | 0.0936 | 6.333e^-07^ | 0.1082 | 0.1082 | 0.0020 |
|  |  |  |  |  |  |  |
| **Flav** | 0.8190 | 0 | 0 | 0 | 0 | 4.096e^-10^ |
|  |  |  |  |  |  |  |
| **Anth** | 3.378e^-06^ | 0.2045 | 0.0289 | 2.175e^-08^ | 0.0171 | 0.0013 |
|  |  |  |  |  |  |  |
| **DUALEX Day 14 of UV treatment – Light backgrounds contrasts** | | | | | | |
|  | | | | | | |
|  | **White × Blue** | **White × Green** | **White × Red** | **Blue × Green** | **Blue × Red** | **Red × Green** |
| **Chl** | 0.0076 | 0.0050 | 9.074e^-07^ | 0.7303 | 0.0050 | 0.0076 |
|  |  |  |  |  |  |  |
| **Flav** | 0.0787 | 1.493e^-11^ | 1.461e^-11^ | 7.113e^-10^ | 7.113e^-10^ | 0.8988 |
|  |  |  |  |  |  |  |
| **Anth** | 0.0258 | 0.2920 | 4.677e^-5^ | 0.2920 | 0.0494 | 0.0017 |
