# Supplementary material for: Spectral Composition of Light Affects Sensitivity to UV-B and Photoinhibition in Cucumber
Source: Front Plant Sci. 2021 Jan 5;11:610011. doi: 10.3389/fpls.2020.610011 (PMC7813804; doi:10.3389/fpls.2020.610011)
Supplement: Supplementary file 4 [file Table_4.DOCX]

|  |  |  |  |  |  |  |
| --- | --- | --- | --- | --- | --- | --- |

**Supplementary Table S4**. Summary of the ANOVA models used to estimate significant main effects of supplementary UV combined with white, blue, green or red light backgrounds on plant biomass (destructive harvest), photosynthesis, chlorophyll fluorescence and epidermal pigment content after 6h of exposure for 14 days.

df_num_ (numerator degrees of freedom), df_den_ (denominator degrees of freedom)

| **Destructive harvest Day 14 of UV treatment** |  |  |  |  |  |
| --- | --- | --- | --- | --- | --- |
| **White vs White + UV** |  |  |  |  |  |
| **Source** | **df_num_** | **df_den_** | ***F*** | ***P*** |  |
| **Height** |  |  |  |  |  |
| Treatment | 1 | 38 | 31.0265 | <0.0001 |  |
|  |  |  |  |  |  |
| **INL** |  |  |  |  |  |
| Treatment | 1 | 38 | 11.054 | 0.002 |  |
|  |  |  |  |  |  |
| **Stem Ø** |  |  |  |  |  |
| Treatment | 1 | 38 | 6.725 | 0.0134 |  |
|  |  |  |  |  |  |
| **LFM** |  |  |  |  |  |
| Treatment | 1 | 38 | 1.376 | 0.2480 |  |
|  |  |  |  |  |  |
| **SFM** |  |  |  |  |  |
| Treatment | 1 | 38 | 34.621 | <0.0001 |  |
|  |  |  |  |  |  |
| **TFM** |  |  |  |  |  |
| Treatment | 1 | 38 | 4.3238 | 0.0444 |  |
|  |  |  |  |  |  |
| **LDM** |  |  |  |  |  |
| Treatment | 1 | 38 | 2.0796 | 0.1575 |  |
|  |  |  |  |  |  |
| **SDM** |  |  |  |  |  |
| Treatment | 1 | 38 | 37.0118 | <0.0001 |  |
|  |  |  |  |  |  |
| **TDM** |  |  |  |  |  |
| Treatment | 1 | 38 | 8.2919 | 0.0065 |  |
|  |  |  |  |  |  |
| **Leaf no** |  |  |  |  |  |
| Treatment | 1 | 38 | 12.294 | 0.0012 |  |
|  |  |  |  |  |  |
| **TLA** |  |  |  |  |  |
| Treatment | 1 | 38 | 12.263 | 0.0012 |  |
|  |  |  |  |  |  |
| **ILA** |  |  |  |  |  |
| Treatment | 1 | 38 | 0.06049 | 0.807 |  |
|  |  |  |  |  |  |
| **DM%** |  |  |  |  |  |
| Treatment | 1 | 38 | 1.0514 | 0.3117 |  |
|  |  |  |  |  |  |
| **SLM** |  |  |  |  |  |
| Treatment | 1 | 38 | 0.50685 | 0.4809 |  |
|  |  |  |  |  |  |
| **LMR** |  |  |  |  |  |
| Treatment | 1 | 38 | 36.9155 | <0.0001 |  |
|  |  |  |  |  |  |
| **Blue vs Blue + UV** |  |  |  |  |  |
| **Source** | **df_num_** | **df_den_** | ***F*** | ***P*** |  |
| **Height** |  |  |  |  |  |
| Treatment | 1 | 38 | 40.984 | <0.0001 |  |
|  |  |  |  |  |  |
| **INL** |  |  |  |  |  |
| Treatment | 1 | 38 | 7.263 | 0.0104 |  |
|  |  |  |  |  |  |
| **Stem Ø** |  |  |  |  |  |
| Treatment | 1 | 38 | 8.3301 | 0.0064 |  |
|  |  |  |  |  |  |
| **LFM** |  |  |  |  |  |
| Treatment | 1 | 38 | 5.051 | 0.0305 |  |
|  |  |  |  |  |  |
| **SFM** |  |  |  |  |  |
| Treatment | 1 | 38 | 60.187 | <0.0001 |  |
|  |  |  |  |  |  |
| **TFM** |  |  |  |  |  |
| Treatment | 1 | 38 | 39.0414 | <0.0001 |  |
|  |  |  |  |  |  |
| **LDM** |  |  |  |  |  |
| Treatment | 1 | 38 | 16.815 | 0.0002 |  |
|  |  |  |  |  |  |
| **SDM** |  |  |  |  |  |
| Treatment | 1 | 38 | 68.181 | <0.0001 |  |
|  |  |  |  |  |  |
| **TDM** |  |  |  |  |  |
| Treatment | 1 | 38 | 38.782 | <0.0001 |  |
|  |  |  |  |  |  |
| **Leaf no** |  |  |  |  |  |
| Treatment | 1 | 38 | 9.452 | 0.0039 |  |
|  |  |  |  |  |  |
| **TLA** |  |  |  |  |  |
| Treatment | 1 | 38 | 15.093 | 0.0004 |  |
|  |  |  |  |  |  |
| **ILA** |  |  |  |  |  |
| Treatment | 1 | 38 | 0.09683 | 0.7574 |  |
|  |  |  |  |  |  |
| **DM%** |  |  |  |  |  |
| Treatment | 1 | 38 | 0.169 | 0.6833 |  |
|  |  |  |  |  |  |
| **SLM** |  |  |  |  |  |
| Treatment | 1 | 38 | 0.4889 | 0.4887 |  |
|  |  |  |  |  |  |
| **LMR** |  |  |  |  |  |
| Treatment | 1 | 38 | 63.8150 | <0.0001 |  |
|  |  |  |  |  |  |
| **Green vs Green + UV** |  |  |  |  |  |
| **Source** | **df_num_** | **df_den_** | ***F*** | ***P*** |  |
| **Height** |  |  |  |  |  |
| Treatment | 1 | 38 | 53.413 | <0.0001 |  |
|  |  |  |  |  |  |
| **INL** |  |  |  |  |  |
| Treatment | 1 | 38 | 30.958 | <0.0001 |  |
|  |  |  |  |  |  |
| **Stem Ø** |  |  |  |  |  |
| Treatment | 1 | 38 | 7.0011 | 0.0118 |  |
|  |  |  |  |  |  |
| **LFM** |  |  |  |  |  |
| Treatment | 1 | 38 | 7.139 | 0.011 |  |
|  |  |  |  |  |  |
| **SFM** |  |  |  |  |  |
| Treatment | 1 | 38 | 34.097 | <0.0001 |  |
|  |  |  |  |  |  |
| **TFM** |  |  |  |  |  |
| Treatment | 1 | 38 | 19.094 | 0.0001 |  |
|  |  |  |  |  |  |
| **LDM** |  |  |  |  |  |
| Treatment | 1 | 38 | 10.2924 | 0.0027 |  |
|  |  |  |  |  |  |
| **SDM** |  |  |  |  |  |
| Treatment | 1 | 38 | 23.827 | <0.0001 |  |
|  |  |  |  |  |  |
| **TDM** |  |  |  |  |  |
| Treatment | 1 | 38 | 15.231 | 0.0004 |  |
|  |  |  |  |  |  |
| **Leaf no** |  |  |  |  |  |
| Treatment | 1 | 38 | 12.5393 | 0.0011 |  |
|  |  |  |  |  |  |
| **TLA** |  |  |  |  |  |
| Treatment | 1 | 38 | 14.151 | 0.0006 |  |
|  |  |  |  |  |  |
| **ILA** |  |  |  |  |  |
| Treatment | 1 | 38 | 3.164 | 0.0833 |  |
|  |  |  |  |  |  |
| **DM%** |  |  |  |  |  |
| Treatment | 1 | 38 | 6.157 | 0.0176 |  |
|  |  |  |  |  |  |
| **SLM** |  |  |  |  |  |
| Treatment | 1 | 38 | 6.5351 | 0.0147 |  |
|  |  |  |  |  |  |
| **LMR** |  |  |  |  |  |
| Treatment | 1 | 38 | 14.62 | 0.0005 |  |
|  |  |  |  |  |  |
| **Red vs Red + UV** |  |  |  |  |  |
| **Source** | **df_num_** | **df_den_** | ***F*** | ***P*** |  |
| **Height** |  |  |  |  |  |
| Treatment | 1 | 38 | 16.253 | 0.0003 |  |
|  |  |  |  |  |  |
| **INL** |  |  |  |  |  |
| Treatment | 1 | 38 | 10.933 | 0.0021 |  |
|  |  |  |  |  |  |
| **Stem Ø** |  |  |  |  |  |
| Treatment | 1 | 38 | 0.36302 | 0.5504 |  |
|  |  |  |  |  |  |
| **LFM** |  |  |  |  |  |
| Treatment | 1 | 38 | 0.4020 | 0.5298 |  |
|  |  |  |  |  |  |
| **SFM** |  |  |  |  |  |
| Treatment | 1 | 38 | 5.658 | 0.0225 |  |
|  |  |  |  |  |  |
| **TFM** |  |  |  |  |  |
| Treatment | 1 | 38 | 0.1159 | 0.7353 |  |
|  |  |  |  |  |  |
| **LDM** |  |  |  |  |  |
| Treatment | 1 | 38 | 0.9184 | 0.3439 |  |
|  |  |  |  |  |  |
| **SDM** |  |  |  |  |  |
| Treatment | 1 | 38 | 0.82677 | 0.3689 |  |
|  |  |  |  |  |  |
| **TDM** |  |  |  |  |  |
| Treatment | 1 | 38 | 0.3665 | 0.5485 |  |
|  |  |  |  |  |  |
| **Leaf no** |  |  |  |  |  |
| Treatment | 1 | 38 | 4.371 | 0.0433 |  |
|  |  |  |  |  |  |
| **TLA** |  |  |  |  |  |
| Treatment | 1 | 38 | 1.1981 | 0.2806 |  |
|  |  |  |  |  |  |
| **ILA** |  |  |  |  |  |
| Treatment | 1 | 38 | 0.06941 | 0.7936 |  |
|  |  |  |  |  |  |
| **DM%** |  |  |  |  |  |
| Treatment | 1 | 38 | 18.545 | 0.0001 |  |
|  |  |  |  |  |  |
| **SLM** |  |  |  |  |  |
| Treatment | 1 | 38 | 30.6192 | <0.0001 |  |
|  |  |  |  |  |  |
| **LMR** |  |  |  |  |  |
| Treatment | 1 | 38 | 20.154 | 0.0004 |  |
|  |  |  |  |  |  |
| **Gas exchange Day 9 – 12 of UV treatment** |  |  |  |  |  |
| **White vs White + UV** |  |  |  |  |  |
| **Source** | **df_num_** | **df_den_** | ***F*** | ***P*** |  |
| **R_dark_** |  |  |  |  |  |
| Treatment | 1 | 38 | 0.01591 | 0.9003 |  |
|  |  |  |  |  |  |
| **α** |  |  |  |  |  |
| Treatment | 1 | 38 | 3.8567 | 0.0569 |  |
|  |  |  |  |  |  |
| **LCP** |  |  |  |  |  |
| Treatment | 1 | 38 | 1.1660 | 0.287 |  |
|  |  |  |  |  |  |
| **Amax** |  |  |  |  |  |
| Treatment | 1 | 38 | 2.4437 | 0.1263 |  |
|  |  |  |  |  |  |
| **θ** |  |  |  |  |  |
| Treatment | 1 | 38 | 5.73127 | 0.0217 |  |
|  |  |  |  |  |  |
| **Blue vs Blue+ UV** |  |  |  |  |  |
| **Source** | **df_num_** | **df_den_** | ***F*** | ***P*** |  |
| **R_dark_** |  |  |  |  |  |
| Treatment | 1 | 38 | 0.04264 | 0.8375 |  |
|  |  |  |  |  |  |
| **α** |  |  |  |  |  |
| Treatment | 1 | 38 | 5.3667 | 0.026 |  |
|  |  |  |  |  |  |
| **LCP** |  |  |  |  |  |
| Treatment | 1 | 38 | 1.9511 | 0.1706 |  |
|  |  |  |  |  |  |
| **Amax** |  |  |  |  |  |
| Treatment | 1 | 38 | 0.22048 | 0.6414 |  |
|  |  |  |  |  |  |
| **θ** |  |  |  |  |  |
| Treatment | 1 | 38 | 0.62199 | 0.4352 |  |
|  |  |  |  |  |  |
| **Green vs Green+ UV** |  |  |  |  |  |
| **Source** | **df_num_** | **df_den_** | ***F*** | ***P*** |  |
| **R_dark_** |  |  |  |  |  |
| Treatment | 1 | 38 | 1.80837 | 0.1867 |  |
|  |  |  |  |  |  |
| **α** |  |  |  |  |  |
| Treatment | 1 | 38 | 2.07819 | 0.1576 |  |
|  |  |  |  |  |  |
| **LCP** |  |  |  |  |  |
| Treatment | 1 | 38 | 0.25485 | 0.6166 |  |
|  |  |  |  |  |  |
| **Amax** |  |  |  |  |  |
| Treatment | 1 | 38 | 0.6505 | 0.425 |  |
|  |  |  |  |  |  |
| **θ** |  |  |  |  |  |
| Treatment | 1 | 38 | 1.8316 | 0.1839 |  |
|  |  |  |  |  |  |
| **Red vs Red+ UV** |  |  |  |  |  |
| **Source** | **df_num_** | **df_den_** | ***F*** | ***P*** |  |
| **R_dark_** |  |  |  |  |  |
| Treatment | 1 | 38 | 7.94438 | 0.0076 |  |
|  |  |  |  |  |  |
| **α** |  |  |  |  |  |
| Treatment | 1 | 38 | 1.62969 | 0.2095 |  |
|  |  |  |  |  |  |
| **LCP** |  |  |  |  |  |
| Treatment | 1 | 38 | 6.93992 | 0.0121 |  |
|  |  |  |  |  |  |
| **Amax** |  |  |  |  |  |
| Treatment | 1 | 38 | 8.21292 | 0.0067 |  |
|  |  |  |  |  |  |
| **θ** |  |  |  |  |  |
| Treatment | 1 | 38 | 7.4213 | 0.0097 |  |

| **CF prior to photoinhibitory treatment – UV treated plants** |  |  |  |  |  |
| --- | --- | --- | --- | --- | --- |
| **White vs White + UV** |  |  |  |  |  |
| **Source** | **df_num_** | **df_den_** | ***F*** | ***P*** |  |
| ***F_v_/F_m_*** |  |  |  |  |  |
| Treatment | 1 | 38 | 8.152 | 0.0069 |  |
|  |  |  |  |  |  |
| **ETR** |  |  |  |  |  |
| Treatment | 1 | 38 | 4.673 | 0.037 |  |
|  |  |  |  |  |  |
| **NPQ** |  |  |  |  |  |
| Treatment | 1 | 38 | 0.4025 | 0.5296 |  |
|  |  |  |  |  |  |
| **q_L_** |  |  |  |  |  |
| Treatment | 1 | 38 | 4.212 | 0.0471 |  |
|  |  |  |  |  |  |
| **Blue vs Blue+ UV** |  |  |  |  |  |
| **Source** | **df_num_** | **df_den_** | ***F*** | ***P*** |  |
| ***F_v_/F_m_*** |  |  |  |  |  |
| Treatment | 1 | 38 | 0.412 | 0.5249 |  |
|  |  |  |  |  |  |
| **ETR** |  |  |  |  |  |
| Treatment | 1 | 38 | 12.022 | 0.0013 |  |
|  |  |  |  |  |  |
| **NPQ** |  |  |  |  |  |
| Treatment | 1 | 38 | 6.640 | 0.014 |  |
|  |  |  |  |  |  |
| **q_L_** |  |  |  |  |  |
| Treatment | 1 | 38 | 13.717 | 0.0007 |  |
|  |  |  |  |  |  |
| **Green vs Green+ UV** |  |  |  |  |  |
| **Source** | **df_num_** | **df_den_** | ***F*** | ***P*** |  |
| ***F_v_/F_m_*** |  |  |  |  |  |
| Treatment | 1 | 38 | 0.0 | 0.9875 |  |
|  |  |  |  |  |  |
| **ETR** |  |  |  |  |  |
| Treatment | 1 | 38 | 0.004 | 0.9481 |  |
|  |  |  |  |  |  |
| **NPQ** |  |  |  |  |  |
| Treatment | 1 | 38 | 11.485 | 0.0016 |  |
|  |  |  |  |  |  |
| **q_L_** |  |  |  |  |  |
| Treatment | 1 | 38 | 0.291 | 0.5929 |  |
|  |  |  |  |  |  |
| **Red vs Red+ UV** |  |  |  |  |  |
| **Source** | **df_num_** | **df_den_** | ***F*** | ***P*** |  |
| ***F_v_/F_m_*** |  |  |  |  |  |
| Treatment | 1 | 38 | 0.294 | 0.5908 |  |
|  |  |  |  |  |  |
| **ETR** |  |  |  |  |  |
| Treatment | 1 | 38 | 3.624 | 0.0646 |  |
|  |  |  |  |  |  |
| **NPQ** |  |  |  |  |  |
| Treatment | 1 | 38 | 0.260 | 0.613 |  |
|  |  |  |  |  |  |
| **q_L_** |  |  |  |  |  |
| Treatment | 1 | 38 | 5.933 | 0.0197 |  |
| **CF after photoinhibitory treatment – UV treated plants** |  |  |  |  |  |
| **White vs White + UV** |  |  |  |  |  |
| **Source** | **df_num_** | **df_den_** | ***F*** | ***P*** |  |
| ***F_v_/F_m_*** |  |  |  |  |  |
| Treatment | 1 | 26 | 5.765 | 0.0238 |  |
|  |  |  |  |  |  |
| **ETR** |  |  |  |  |  |
| Treatment | 1 | 26 | 0.047 | 0.8294 |  |
|  |  |  |  |  |  |
| **NPQ** |  |  |  |  |  |
| Treatment | 1 | 26 | 9.414 | 0.005 |  |
|  |  |  |  |  |  |
| **q_L_** |  |  |  |  |  |
| Treatment | 1 | 26 | 0.42284 | 0.5212 |  |
|  |  |  |  |  |  |
| **Blue vs Blue+ UV** |  |  |  |  |  |
| **Source** | **df_num_** | **df_den_** | ***F*** | ***P*** |  |
| ***F_v_/F_m_*** |  |  |  |  |  |
| Treatment | 1 | 25 | 22.217 | 0.0001 |  |
|  |  |  |  |  |  |
| **ETR** |  |  |  |  |  |
| Treatment | 1 | 25 | 24.884 | <0.0001 |  |
|  |  |  |  |  |  |
| **NPQ** |  |  |  |  |  |
| Treatment | 1 | 25 | 3.848 | 0.061 |  |
|  |  |  |  |  |  |
| **q_L_** |  |  |  |  |  |
| Treatment | 1 | 25 | 15.277 | 0.0006 |  |
|  |  |  |  |  |  |
| **Green vs Green+ UV** |  |  |  |  |  |
| **Source** | **df_num_** | **df_den_** | ***F*** | ***P*** |  |
| ***F_v_/F_m_*** |  |  |  |  |  |
| Treatment | 1 | 26 | 4.446 | 0.0448 |  |
|  |  |  |  |  |  |
| **ETR** |  |  |  |  |  |
| Treatment | 1 | 26 | 1.048 | 0.3153 |  |
|  |  |  |  |  |  |
| **NPQ** |  |  |  |  |  |
| Treatment | 1 | 26 | 3.16666 | 0.0868 |  |
|  |  |  |  |  |  |
| **q_L_** |  |  |  |  |  |
| Treatment | 1 | 26 | 0.33884 | 0.5655 |  |
|  |  |  |  |  |  |
| **Red vs Red+ UV** |  |  |  |  |  |
| **Source** | **df_num_** | **df_den_** | ***F*** | ***P*** |  |
| ***F_v_/F_m_*** |  |  |  |  |  |
| Treatment | 1 | 26 | 0.026 | 0.8723 |  |
|  |  |  |  |  |  |
| **ETR** |  |  |  |  |  |
| Treatment | 1 | 26 | 7.386 | 0.0115 |  |
|  |  |  |  |  |  |
| **NPQ** |  |  |  |  |  |
| Treatment | 1 | 26 | 0.028 | 0.8676 |  |
|  |  |  |  |  |  |
| **q_L_** |  |  |  |  |  |
| Treatment | 1 | 26 | 4.557 | 0.0424 |  |

| **Before Photoinhibitory treatment vs After photoinhibitory treatment** |  |  |  |  |  |
| --- | --- | --- | --- | --- | --- |
| **White BPI vs White PI** |  |  |  |  |  |
| **Source** | **df_num_** | **df_den_** | ***F*** | ***P*** |  |
| ***F_v_/F_m_*** |  |  |  |  |  |
| Treatment | 1 | 14 | 15.633 | 0.0014 |  |
|  |  |  |  |  |  |
| **ETR** |  |  |  |  |  |
| Treatment | 1 | 14 | 1.82861 | 0.1977 |  |
|  |  |  |  |  |  |
| **NPQ** |  |  |  |  |  |
| Treatment | 1 | 14 | 0.20653 | 0.6565 |  |
|  |  |  |  |  |  |
| **q_L_** |  |  |  |  |  |
| Treatment | 1 | 14 | 0.10214 | 0.754 |  |
|  |  |  |  |  |  |
| **White UV BPI vs White UV PI** |  |  |  |  |  |
| **Source** | **df_num_** | **df_den_** | ***F*** | ***P*** |  |
| ***F_v_/F_m_*** |  |  |  |  |  |
| Treatment | 1 | 14 | 5.3793 | 0.036 |  |
|  |  |  |  |  |  |
| **ETR** |  |  |  |  |  |
| Treatment | 1 | 14 | 0.79567 | 0.3875 |  |
|  |  |  |  |  |  |
| **NPQ** |  |  |  |  |  |
| Treatment | 1 | 14 | 4.98699 | 0.0424 |  |
|  |  |  |  |  |  |
| **q_L_** |  |  |  |  |  |
| Treatment | 1 | 14 | 0.77418 | 0.3938 |  |
|  |  |  |  |  |  |
| **Blue BPI vs Blue PI** |  |  |  |  |  |
| **Source** | **df_num_** | **df_den_** | ***F*** | ***P*** |  |
| ***F_v_/F_m_*** |  |  |  |  |  |
| Treatment | 1 | 13 | 47.25 | <0.0001 |  |
|  |  |  |  |  |  |
| **ETR** |  |  |  |  |  |
| Treatment | 1 | 13 | 0.1905 | 0.6697 |  |
|  |  |  |  |  |  |
| **NPQ** |  |  |  |  |  |
| Treatment | 1 | 13 | 8.9054 | 0.0106 |  |
|  |  |  |  |  |  |
| **q_L_** |  |  |  |  |  |
| Treatment | 1 | 13 | 1.3501 | 0.2661 |  |
|  |  |  |  |  |  |
| **Blue UV BPI vs Blue UV PI** |  |  |  |  |  |
| **Source** | **df_num_** | **df_den_** | ***F*** | ***P*** |  |
| ***F_v_/F_m_*** |  |  |  |  |  |
| Treatment | 1 | 14 | 97.245 | <0.0001 |  |
|  |  |  |  |  |  |
| **ETR** |  |  |  |  |  |
| Treatment | 1 | 14 | 10.238 | 0.0064 |  |
|  |  |  |  |  |  |
| **NPQ** |  |  |  |  |  |
| Treatment | 1 | 14 | 12.828 | 0.003 |  |
|  |  |  |  |  |  |
| **q_L_** |  |  |  |  |  |
| Treatment | 1 | 14 | 0.01676 | 0.8988 |  |
|  |  |  |  |  |  |
| **Green BPI vs Green PI** |  |  |  |  |  |
| **Source** | **df_num_** | **df_den_** | ***F*** | ***P*** |  |
| ***F_v_/F_m_*** |  |  |  |  |  |
| Treatment | 1 | 13 | 65.228 | <0.0001 |  |
|  |  |  |  |  |  |
| **ETR** |  |  |  |  |  |
| Treatment | 1 | 13 | 5.171 | 0.0406 |  |
|  |  |  |  |  |  |
| **NPQ** |  |  |  |  |  |
| Treatment | 1 | 13 | 38.627 | <0.0001 |  |
|  |  |  |  |  |  |
| **q_L_** |  |  |  |  |  |
| Treatment | 1 | 13 | 12.894 | 0.0033 |  |
|  |  |  |  |  |  |
| **Green UV BPI vs Green UV PI** |  |  |  |  |  |
| **Source** | **df_num_** | **df_den_** | ***F*** | ***P*** |  |
| ***F_v_/F_m_*** |  |  |  |  |  |
| Treatment | 1 | 15 | 69.772 | <0.0001 |  |
|  |  |  |  |  |  |
| **ETR** |  |  |  |  |  |
| Treatment | 1 | 15 | 0.0694 | 0.7957 |  |
|  |  |  |  |  |  |
| **NPQ** |  |  |  |  |  |
| Treatment | 1 | 15 | 47.0189 | <0.0001 |  |
|  |  |  |  |  |  |
| **q_L_** |  |  |  |  |  |
| Treatment | 1 | 15 | 8.242 | 0.0117 |  |
|  |  |  |  |  |  |
|  |  |  |  |  |  |
| **Red BPI vs Red BPI** |  |  |  |  |  |
| **Source** | **df_num_** | **df_den_** | ***F*** | ***P*** |  |
| ***F_v_/F_m_*** |  |  |  |  |  |
| Treatment | 1 | 14 | 5.3572 | 0.0363 |  |
|  |  |  |  |  |  |
| **ETR** |  |  |  |  |  |
| Treatment | 1 | 14 | 1.11383 | 0.3091 |  |
|  |  |  |  |  |  |
| **NPQ** |  |  |  |  |  |
| Treatment | 1 | 14 | 1.0106 | 0.3318 |  |
|  |  |  |  |  |  |
| **q_L_** |  |  |  |  |  |
| Treatment | 1 | 14 | 7.3300 | 0.017 |  |
|  |  |  |  |  |  |
| **Red UV BPI vs RedUV PI** |  |  |  |  |  |
| **Source** | **df_num_** | **df_den_** | ***F*** | ***P*** |  |
| ***F_v_/F_m_*** |  |  |  |  |  |
| Treatment | 1 | 13 | 7.50225 | 0.0169 |  |
|  |  |  |  |  |  |
| **ETR** |  |  |  |  |  |
| Treatment | 1 | 13 | 10.873 | 0.0058 |  |
|  |  |  |  |  |  |
| **NPQ** |  |  |  |  |  |
| Treatment | 1 | 13 | 2.620 | 0.1295 |  |
|  |  |  |  |  |  |
| **q_L_** |  |  |  |  |  |
| Treatment | 1 | 13 | 9.9563 | 0.0076 |  |

| **DUALEX Day 14 of UV treatment** |  |  |  |  |  |
| --- | --- | --- | --- | --- | --- |
| **White vs White + UV** |  |  |  |  |  |
| **Source** | **df_num_** | **df_den_** | ***F*** | ***P*** |  |
| **Chl** |  |  |  |  |  |
| Treatment | 1 | 20 | 5.57139 | 0.0285 |  |
|  |  |  |  |  |  |
| **Flav** |  |  |  |  |  |
| Treatment | 1 | 20 | 2.0870 | 0.164 |  |
|  |  |  |  |  |  |
| **Anth** |  |  |  |  |  |
| Treatment | 1 | 20 | 3.501357 | 0.076 |  |
|  |  |  |  |  |  |
| **Blue vs Blue+ UV** |  |  |  |  |  |
| **Source** | **df_num_** | **df_den_** | ***F*** | ***P*** |  |
| **Chl** |  |  |  |  |  |
| Treatment | 1 | 20 | 0.44818 | 0.5109 |  |
|  |  |  |  |  |  |
| **Flav** |  |  |  |  |  |
| Treatment | 1 | 20 | 11.95228 | 0.0025 |  |
|  |  |  |  |  |  |
| **Anth** |  |  |  |  |  |
| Treatment | 1 | 20 | 8.91437 | 0.0073 |  |
|  |  |  |  |  |  |
| **Green vs Green+ UV** |  |  |  |  |  |
| **Source** | **df_num_** | **df_den_** | ***F*** | ***P*** |  |
| **Chl** |  |  |  |  |  |
| Treatment | 1 | 20 | 6.1615 | 0.0221 |  |
|  |  |  |  |  |  |
| **Flav** |  |  |  |  |  |
| Treatment | 1 | 20 | 11.19041 | 0.0032 |  |
|  |  |  |  |  |  |
| **Anth** |  |  |  |  |  |
| Treatment | 1 | 20 | 0.68501 | 0.4176 |  |
|  |  |  |  |  |  |
| **Red vs Red+ UV** |  |  |  |  |  |
| **Source** | **df_num_** | **df_den_** | ***F*** | ***P*** |  |
| **Chl** |  |  |  |  |  |
| Treatment | 1 | 20 | 0.0434 | 0.8371 |  |
|  |  |  |  |  |  |
| **Flav** |  |  |  |  |  |
| Treatment | 1 | 20 | 1.10108 | 0.3065 |  |
|  |  |  |  |  |  |
| **Anth** |  |  |  |  |  |
| Treatment | 1 | 20 | 1.65494 | 0.213 |  |
