# Supplementary material for: Spectral Composition of Light Affects Sensitivity to UV-B and Photoinhibition in Cucumber
Source: Front Plant Sci. 2021 Jan 5;11:610011. doi: 10.3389/fpls.2020.610011 (PMC7813804; doi:10.3389/fpls.2020.610011)
Supplement: Supplementary file 5 [file Table_5.DOCX]

**
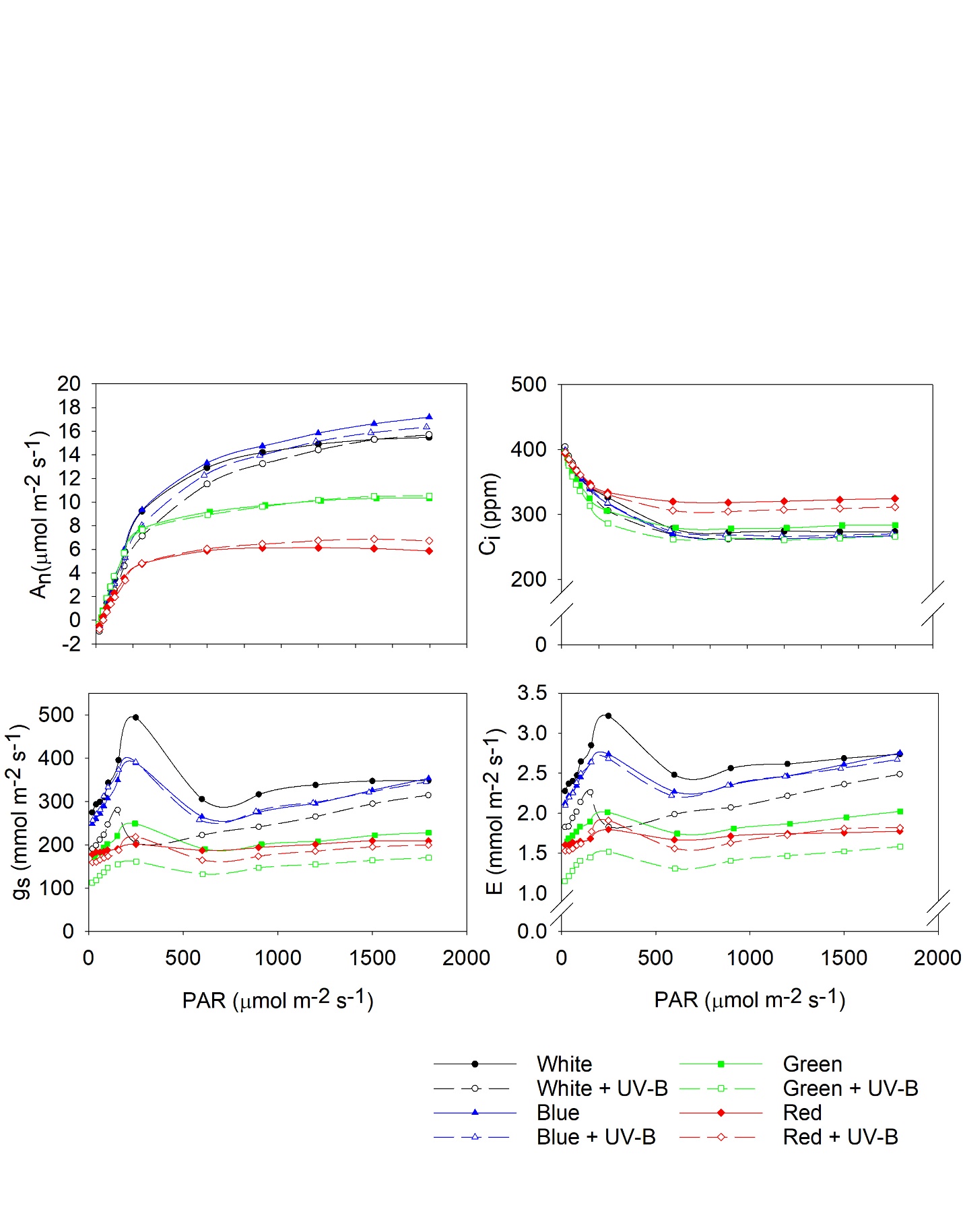
**

**D**

**C**

**B**

**A**

**Supplementary Figure S1.** Data of the light response curves used for Fig. 2A measured at day 9 to 13 of UV-B treatment in control Perspex OTBF boxes (Qian et al., 2019) with no UV-B exposure (solid line) and cellulose Acetate OTBF boxes with UV-B exposure (dashed lines) under four different PAR treatments (broadband white, blue, green and red). (**A**) net photosynthetic rate (P_n_); (**B**) stomatal conductance (g_s_), (**C**) intercellular CO_2_ (C_i_) and (**D**) transpiration rate (E). The data represent mean values (n = 7)
