# Supplementary material for: Spectral Composition of Light Affects Sensitivity to UV-B and Photoinhibition in Cucumber
Source: Front Plant Sci. 2021 Jan 5;11:610011. doi: 10.3389/fpls.2020.610011 (PMC7813804; doi:10.3389/fpls.2020.610011)
Supplement: Supplementary file 6 [file Table_6.DOCX]

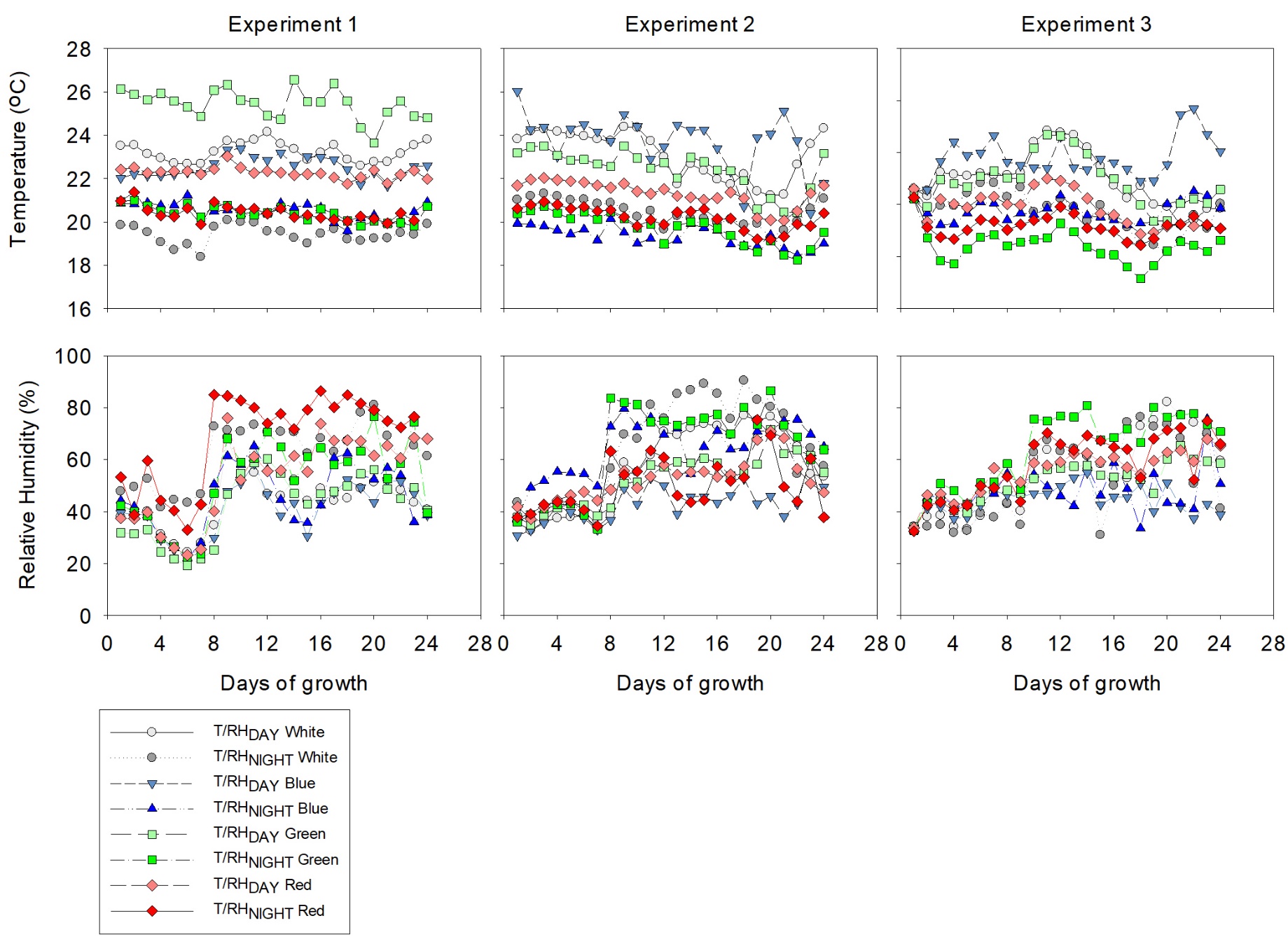


**Supplementary Figure S2.** Mean temperature (°C) and relative humidity (%) measured during the three experimental replicates in four different light treatments (broadband white, blue, green and red). The solid lines represent the OTBF (Qian et al., 2019) control boxes without UV-B and the dashed lines represent the OTBF boxes exposed to UV-B.
