# Supplementary material for: Spectral Composition of Light Affects Sensitivity to UV-B and Photoinhibition in Cucumber
Source: Front Plant Sci. 2021 Jan 5;11:610011. doi: 10.3389/fpls.2020.610011 (PMC7813804; doi:10.3389/fpls.2020.610011)
Supplement: Supplementary file 7 [file Table_7.DOCX]

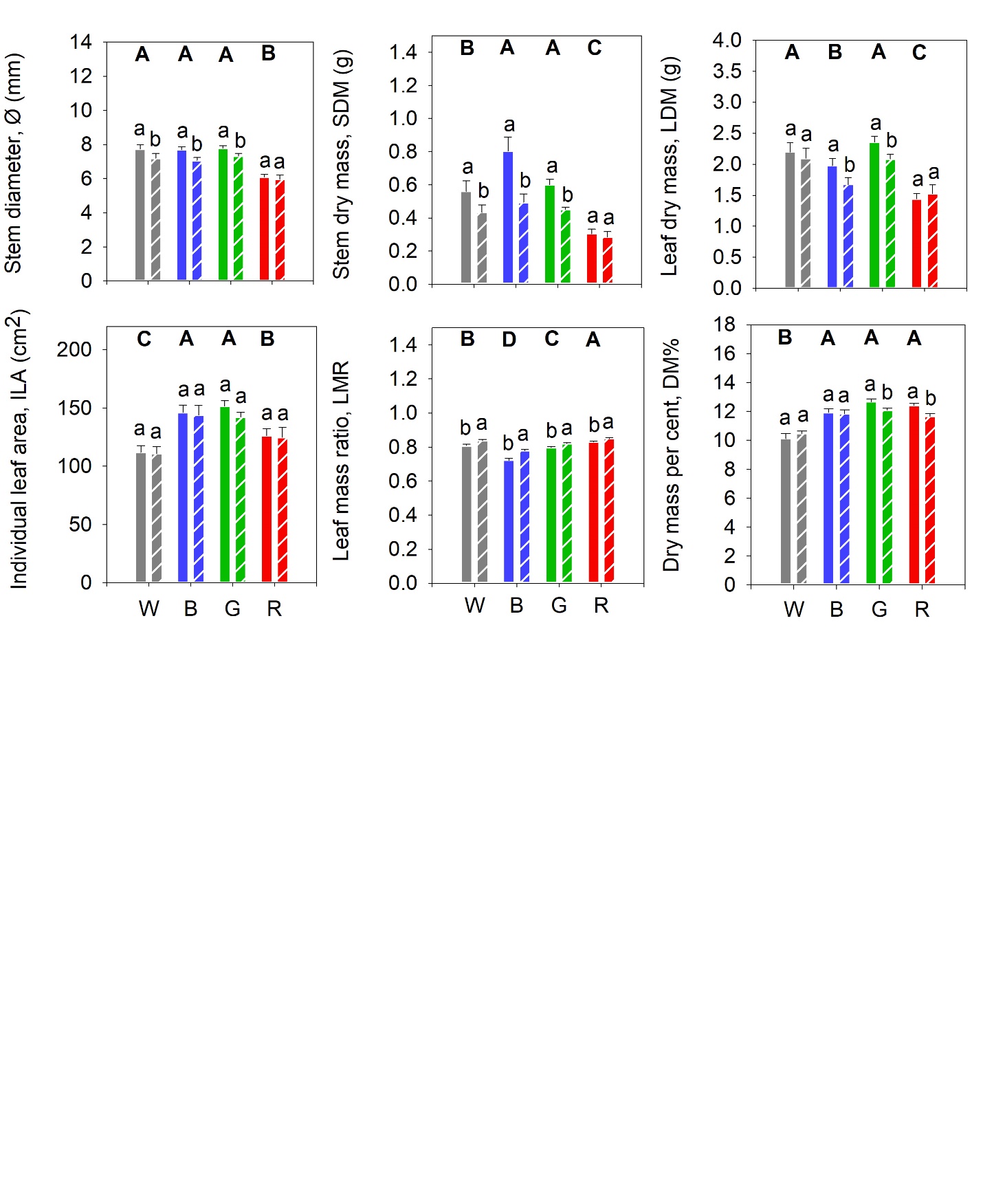


**F**

**E**

**D**

**C**

**B**

**A**

**Supplementary Figure S3.** Biomass accumulation of cucumber plants grown under four PAR backgrounds after 14 days without or with exposure to supplementary UV-B irradiation. (**A**) Stem diameter (Ø, mm); (**B**) Stem dry mass (SDM, g); (**C**) Leaf dry mass (LDM, g); (**D**) Individual leaf area (ILA, cm^2^); (**E**) Leaf mass ratio (LMR); (**F**) Dry mass per cent (DM%, %). Data are mean values (n = 7 ± SE). Capital letters indicate significant difference between growth light qualities without UV-B and lower case letters between non-UV-B-exposed plants and UV-B-exposed plants within the same light backgrounds, both at P < 0.05.
